# Supplementary material for: Estimating global bee species richness and taxonomic gaps
Source: Nat Commun. 2026 Feb 24;17:1762. doi: 10.1038/s41467-026-69029-4 (PMC12932799; doi:10.1038/s41467-026-69029-4)
Supplement: Supplementary file 1 — Supplementary Information [file 41467_2026_69029_MOESM1_ESM.pdf]

Supplementary files to: Estimating global bee species richness and taxonomic gaps

James B. Dorey[1,2,3]*, Amy-Marie Gilpin[4,5], Nikolas Johnson[1,2], Damien Esquerré[1,2], Alice C. Hughes[6], John S. Ascher[7,8], Michael C. Orr[9,10,11]*

[1]Environmental Futures Research Centre, School of Science, University of Wollongong; Wollongong, 2500, Australia.

[2]Molecular Horizons Research Institute, School of Science, University of Wollongong; Wollongong, 2500, Australia.

[3]College of Science and Engineering, Flinders University, Bedford Park, 5042, Australia

[4]School of Science, Western Sydney University; Penrith, 2750, Australia.

[5]Hawkesbury Institute for the Environment, Western Sydney University, Penrith, 2750, Australia

[6]Faculty of Science, University of Hong Kong; Hong Kong, China.

[7]Department of Biological Sciences, National University of Singapore; Singapore, 117543, Singapore.

[8]Lee Kong Chian Natural History Museum, Singapore, 117377, Singapore

[9]Entomologie, Staatliches Museum für Naturkunde Stuttgart; Stuttgart, 70191, Germany.

[10]Institute of Zoology, Chinese Academy of Sciences, Beijing, 100101, China.

[11]KomBioTa-Center for Biodiversity and Integrative Taxonomy, University of Hohenheim and State Museum of Natural History, Stuttgart, 70191, Germany

*Corresponding authors. Email: jbdorey@me.com and michael.christopher.orr@gmail.com

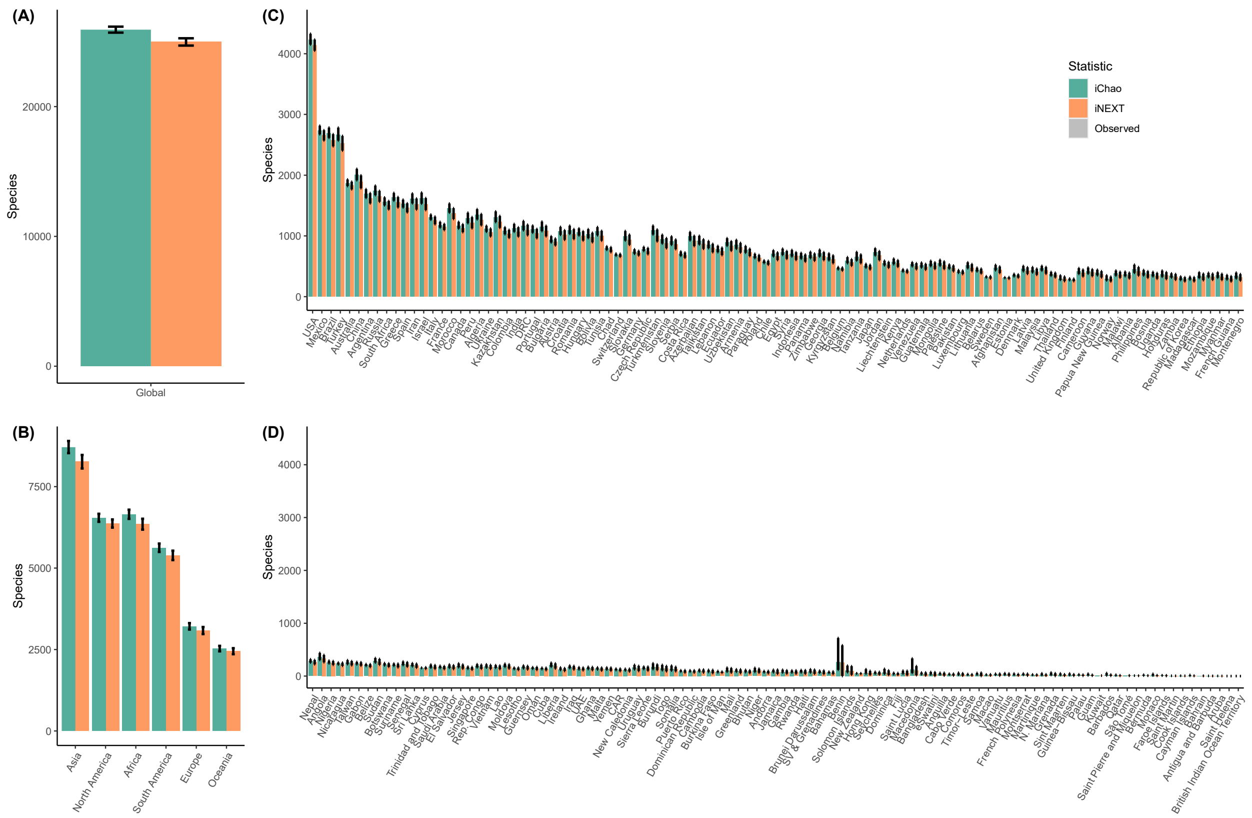

Fig. S1.

The (**A**) global, (**B**) continental, and (**C and D**) country plots showing the number of observed species (grey) and the number of estimated species using the statistics iChao (green) and iNEXT (orange). Estimates are from a single sample of the literature curve combined with the empirical data. Bars indicate 95% confidence intervals. The original publication and original input data can be downloaded from https://doi.org/10.25451/flinders.21709757. Source data are provided as a Source Data file.

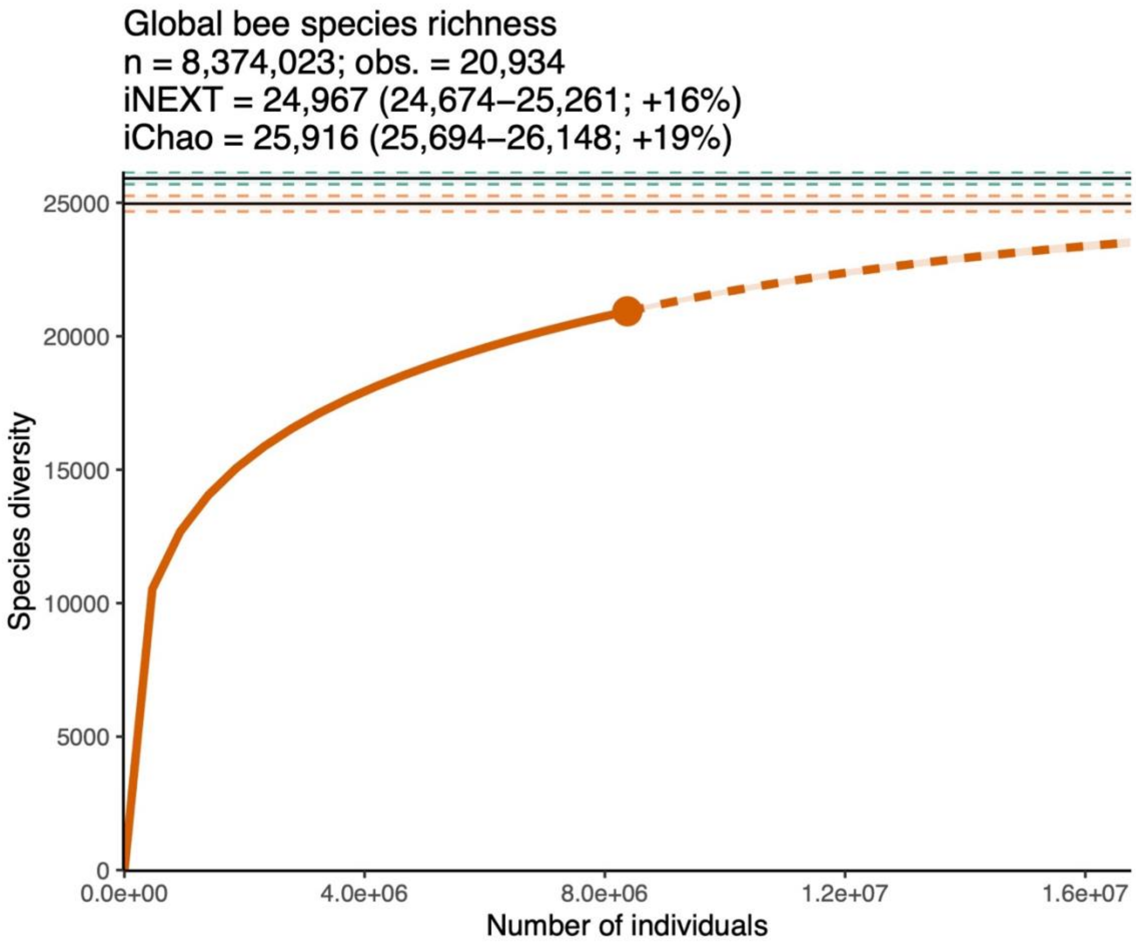

Fig. S2.

The global plot showing the estimated species accumulation curves from iNEXT (orange curve), the estimated value and 95% confidence intervals from iNEXT (orange) and iChao (green) over the number of individuals. The dashed curve indicates the iNEXT extrapolation past 100% of the empirical sample size. Estimates are from a single sample of the literature curve combined with the empirical data. The original publication and original input data can be downloaded from https://doi.org/10.25451/flinders.21709757. Source data are provided as a Source Data file.

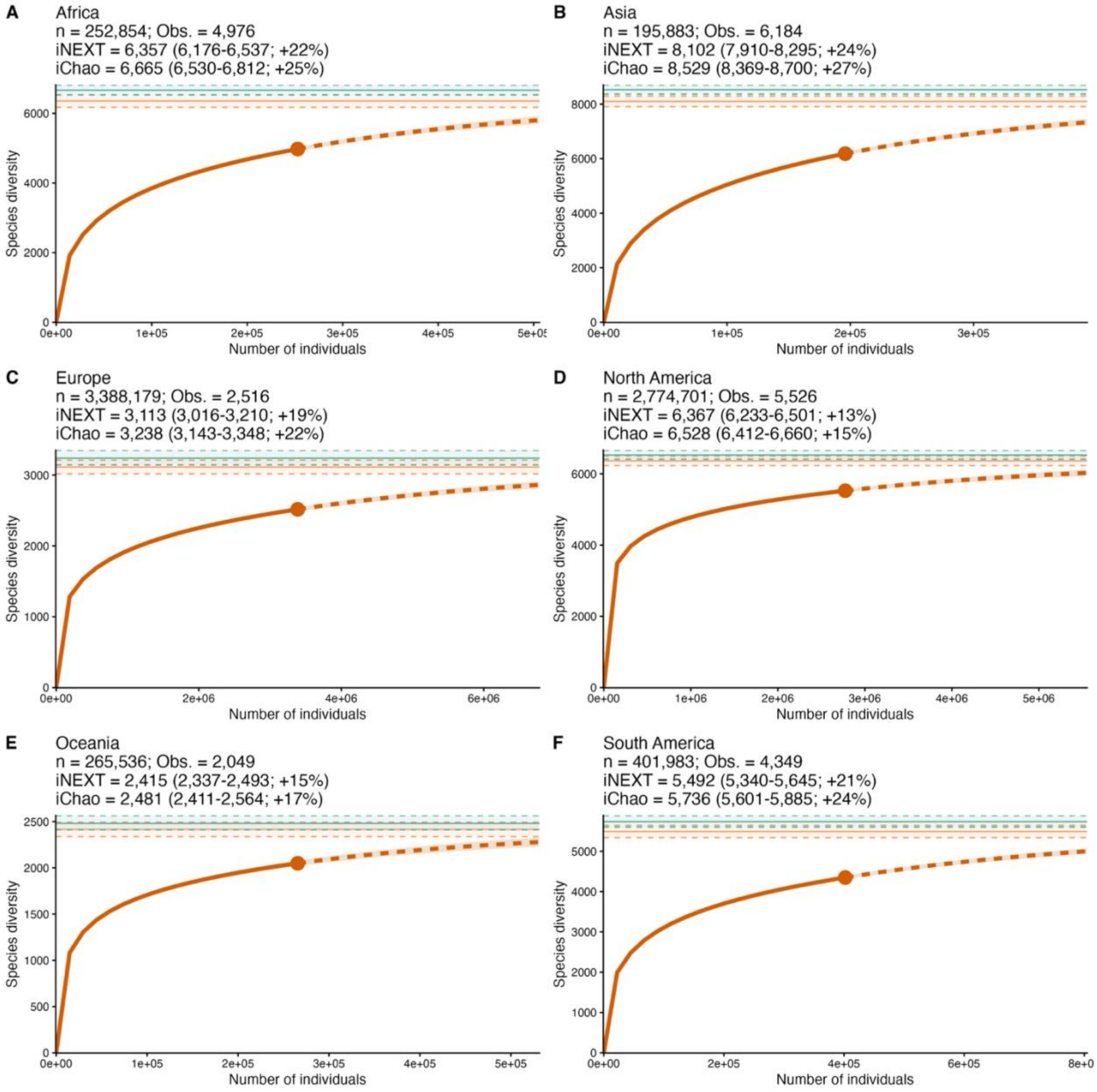

Fig. S3.

The continental plots showing the estimated species accumulation curves from iNEXT (orange curve), the estimated value and 95% confidence intervals from iNEXT (orange) and iChao (green) over the number of individuals. The dashed curve indicates the iNEXT extrapolation past 100% of the empirical sample size. Estimates are from a single sample of the literature curve combined with the empirical data. Estimates are from a single sample of the literature curve combined with the empirical data. The original publication and original input data can be downloaded from https://doi.org/10.25451/flinders.21709757. Source data are provided as a Source Data file.

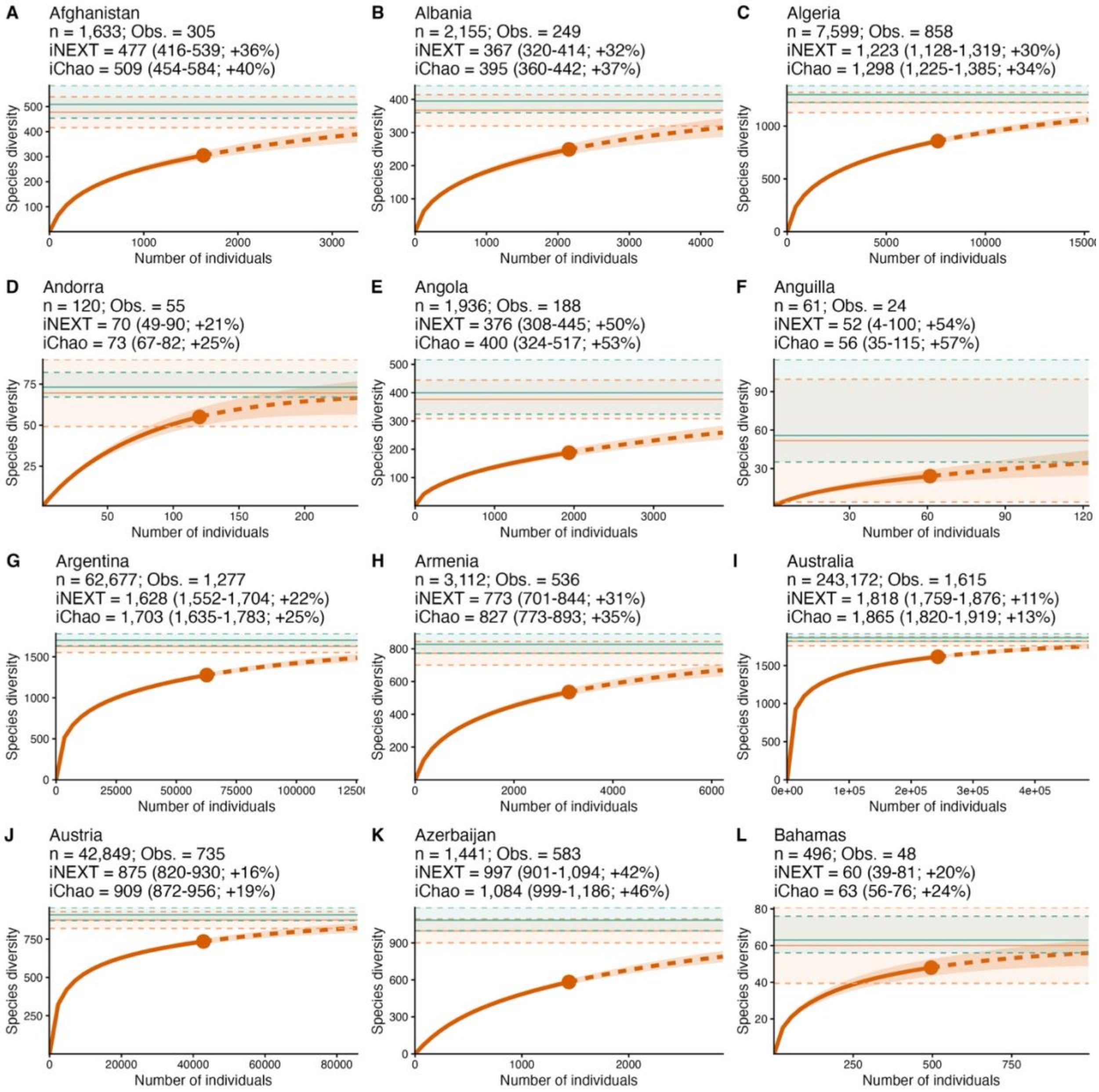
A
Afghanistan
n = 1,633; Obs. = 305
iNEXT = 477 (416-539; +36%)
iChao = 509 (454-584; +40%)
B
Albania
n = 2,155; Obs. = 249
iNEXT = 367 (320-414; +32%)
iChao = 395 (360-442; +37%)
C
Algeria
n = 7,599; Obs. = 858
iNEXT = 1,223 (1,128-1,319; +30%)
iChao = 1,298 (1,225-1,385; +34%)
D
Andorra
n = 120; Obs. = 55
iNEXT = 70 (49-90; +21%)
iChao = 73 (67-82; +25%)
E
Angola
n = 1,936; Obs. = 188
iNEXT = 376 (308-445; +50%)
iChao = 400 (324-517; +53%)
F
Anguilla
n = 61; Obs. = 24
iNEXT = 52 (4-100; +54%)
iChao = 56 (35-115; +57%)
G
Argentina
n = 62,677; Obs. = 1,277
iNEXT = 1,628 (1,552-1,704; +22%)
iChao = 1,703 (1,635-1,783; +25%)
H
Armenia
n = 3,112; Obs. = 536
iNEXT = 773 (701-844; +31%)
iChao = 827 (773-893; +35%)
I
Australia
n = 243,172; Obs. = 1,615
iNEXT = 1,818 (1,759-1,876; +11%)
iChao = 1,865 (1,820-1,919; +13%)
J
Austria
n = 42,849; Obs. = 735
iNEXT = 875 (820-930; +16%)
iChao = 909 (872-956; +19%)
K
Azerbaijan
n = 1,441; Obs. = 583
iNEXT = 997 (901-1,094; +42%)
iChao = 1,084 (999-1,186; +46%)
L
Bahamas
n = 496; Obs. = 48
iNEXT = 60 (39-81; +20%)
iChao = 63 (56-76; +24%)
Species diversity
Number of individuals

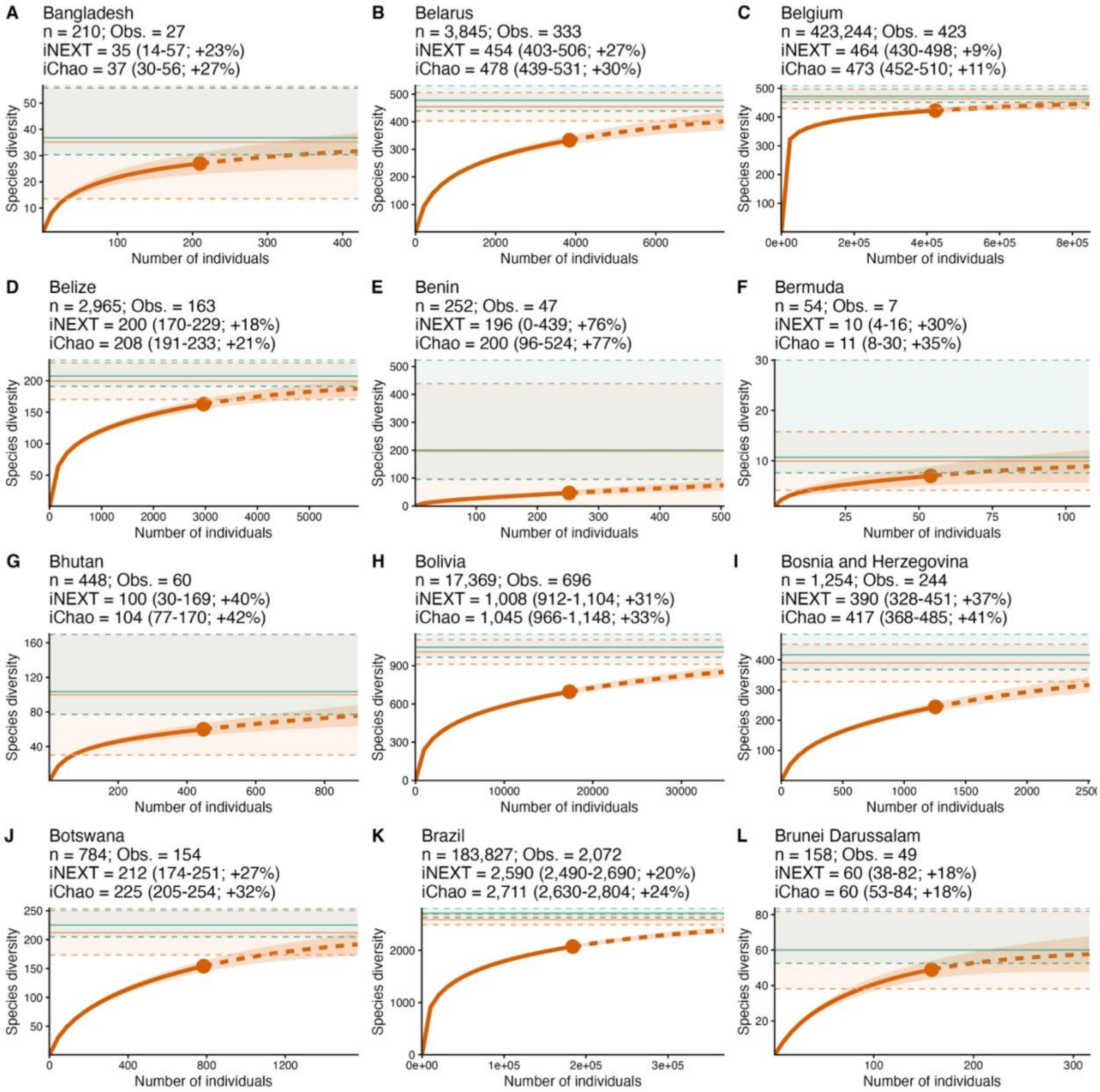
A Bangladesh
n = 210; Obs. = 27
iNEXT = 35 (14-57; +23%)
iChao = 37 (30-56; +27%)
B Belarus
n = 3,845; Obs. = 333
iNEXT = 454 (403-506; +27%)
iChao = 478 (439-531; +30%)
C Belgium
n = 423,244; Obs. = 423
iNEXT = 464 (430-498; +9%)
iChao = 473 (452-510; +11%)
D Belize
n = 2,965; Obs. = 163
iNEXT = 200 (170-229; +18%)
iChao = 208 (191-233; +21%)
E Benin
n = 252; Obs. = 47
iNEXT = 196 (0-439; +76%)
iChao = 200 (96-524; +77%)
F Bermuda
n = 54; Obs. = 7
iNEXT = 10 (4-16; +30%)
iChao = 11 (8-30; +35%)
G Bhutan
n = 448; Obs. = 60
iNEXT = 100 (30-169; +40%)
iChao = 104 (77-170; +42%)
H Bolivia
n = 17,369; Obs. = 696
iNEXT = 1,008 (912-1,104; +31%)
iChao = 1,045 (966-1,148; +33%)
I Bosnia and Herzegovina
n = 1,254; Obs. = 244
iNEXT = 390 (328-451; +37%)
iChao = 417 (368-485; +41%)
J Botswana
n = 784; Obs. = 154
iNEXT = 212 (174-251; +27%)
iChao = 225 (205-254; +32%)
K Brazil
n = 183,827; Obs. = 2,072
iNEXT = 2,590 (2,490-2,690; +20%)
iChao = 2,711 (2,630-2,804; +24%)
L Brunei Darussalam
n = 158; Obs. = 49
iNEXT = 60 (38-82; +18%)
iChao = 60 (53-84; +18%)
Species diversity
Number of individuals

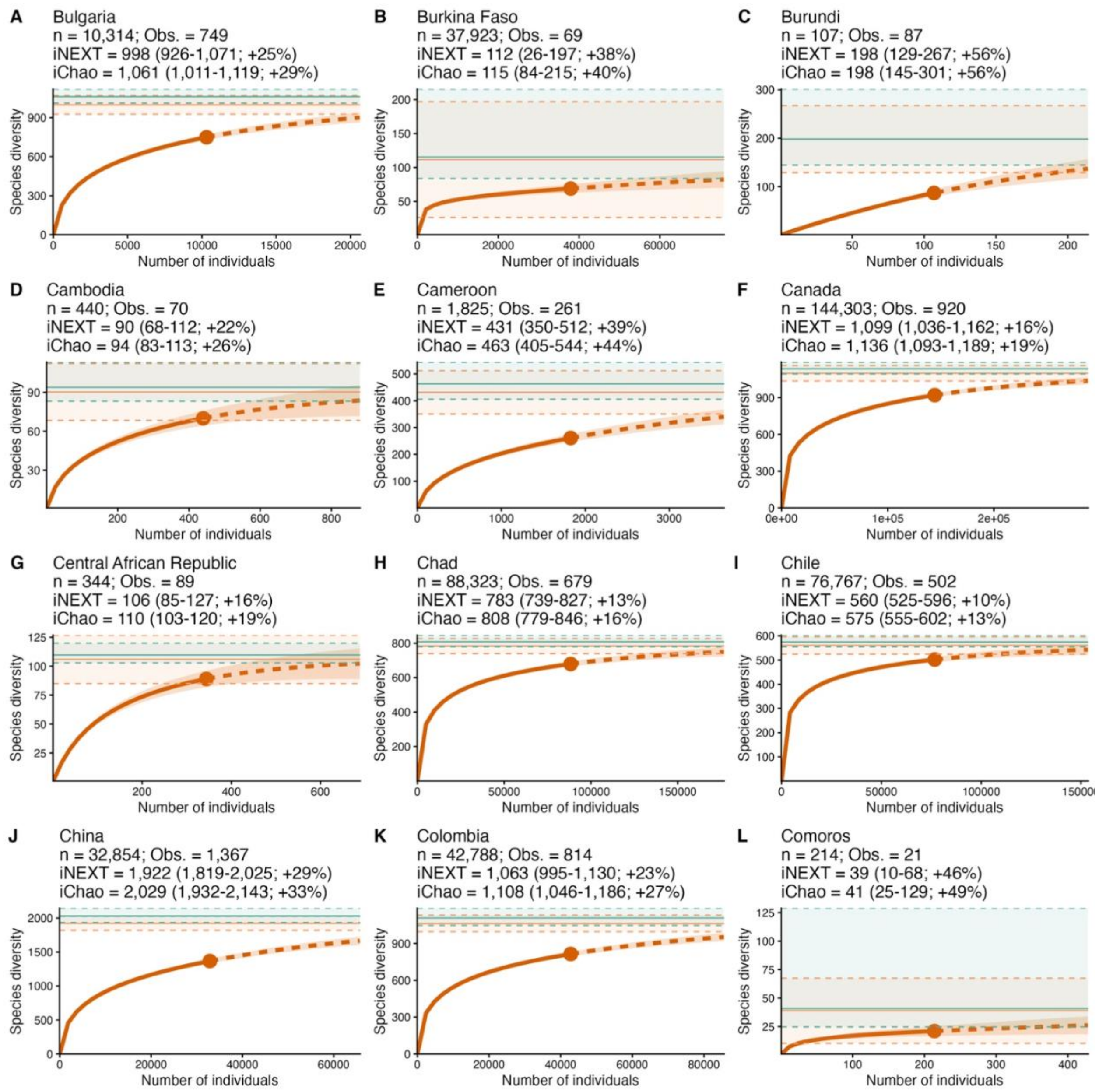
A
Bulgaria
n = 10,314; Obs. = 749
iNEXT = 998 (926-1,071; +25%)
iChao = 1,061 (1,011-1,119; +29%)
B
Burkina Faso
n = 37,923; Obs. = 69
iNEXT = 112 (26-197; +38%)
iChao = 115 (84-215; +40%)
C
Burundi
n = 107; Obs. = 87
iNEXT = 198 (129-267; +56%)
iChao = 198 (145-301; +56%)
D
Cambodia
n = 440; Obs. = 70
iNEXT = 90 (68-112; +22%)
iChao = 94 (83-113; +26%)
E
Cameroon
n = 1,825; Obs. = 261
iNEXT = 431 (350-512; +39%)
iChao = 463 (405-544; +44%)
F
Canada
n = 144,303; Obs. = 920
iNEXT = 1,099 (1,036-1,162; +16%)
iChao = 1,136 (1,093-1,189; +19%)
G
Central African Republic
n = 344; Obs. = 89
iNEXT = 106 (85-127; +16%)
iChao = 110 (103-120; +19%)
H
Chad
n = 88,323; Obs. = 679
iNEXT = 783 (739-827; +13%)
iChao = 808 (779-846; +16%)
I
Chile
n = 76,767; Obs. = 502
iNEXT = 560 (525-596; +10%)
iChao = 575 (555-602; +13%)
J
China
n = 32,854; Obs. = 1,367
iNEXT = 1,922 (1,819-2,025; +29%)
iChao = 2,029 (1,932-2,143; +33%)
K
Colombia
n = 42,788; Obs. = 814
iNEXT = 1,063 (995-1,130; +23%)
iChao = 1,108 (1,046-1,186; +27%)
L
Comoros
n = 214; Obs. = 21
iNEXT = 39 (10-68; +46%)
iChao = 41 (25-129; +49%)
Species diversity
Number of individuals

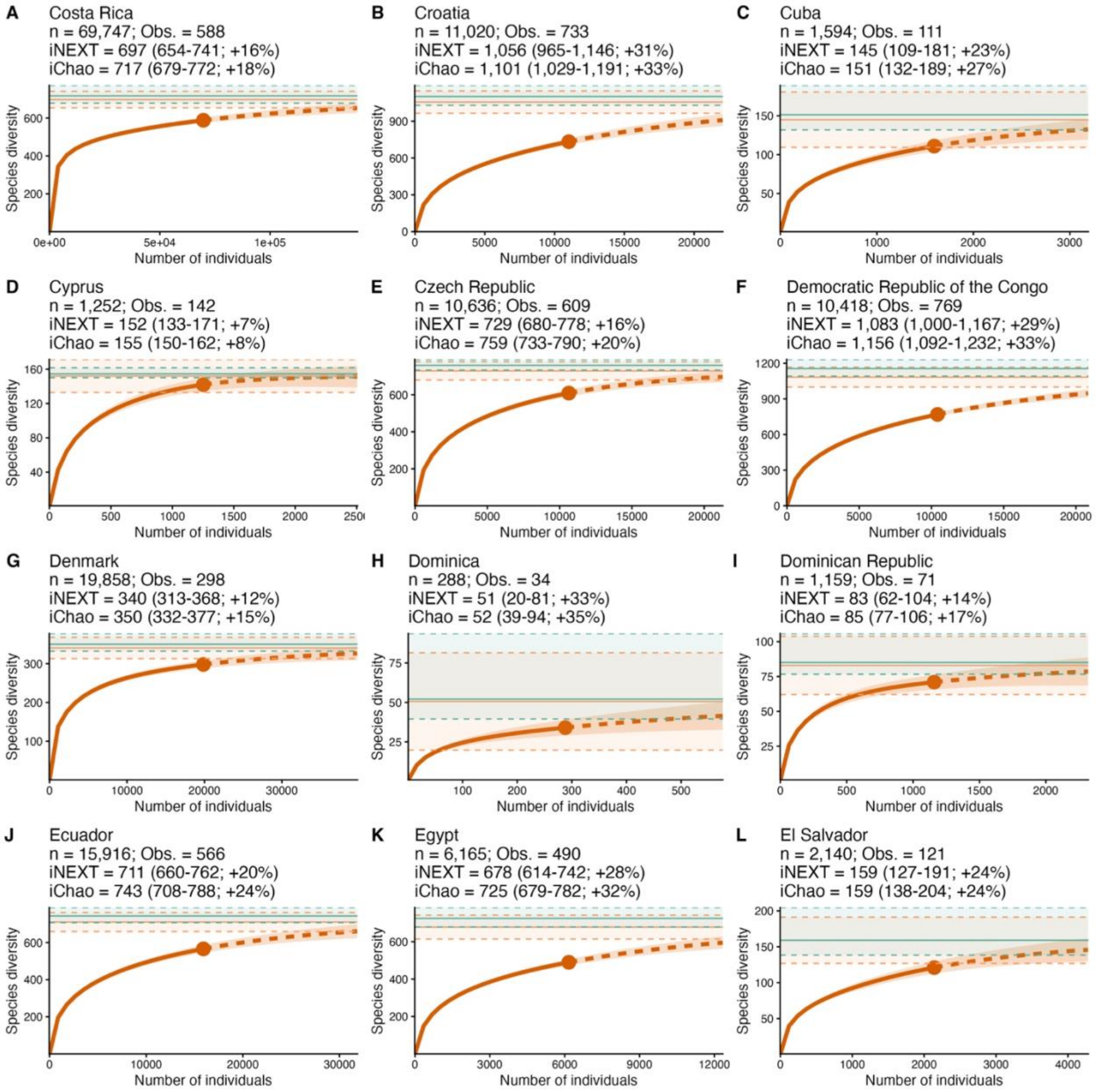
A
Costa Rica
n = 69,747; Obs. = 588
iNEXT = 697 (654-741; +16%)
iChao = 717 (679-772; +18%)
Species diversity
Number of individuals
B
Croatia
n = 11,020; Obs. = 733
iNEXT = 1,056 (965-1,146; +31%)
iChao = 1,101 (1,029-1,191; +33%)
Species diversity
Number of individuals
C
Cuba
n = 1,594; Obs. = 111
iNEXT = 145 (109-181; +23%)
iChao = 151 (132-189; +27%)
Species diversity
Number of individuals
D
Cyprus
n = 1,252; Obs. = 142
iNEXT = 152 (133-171; +7%)
iChao = 155 (150-162; +8%)
Species diversity
Number of individuals
E
Czech Republic
n = 10,636; Obs. = 609
iNEXT = 729 (680-778; +16%)
iChao = 759 (733-790; +20%)
Species diversity
Number of individuals
F
Democratic Republic of the Congo
n = 10,418; Obs. = 769
iNEXT = 1,083 (1,000-1,167; +29%)
iChao = 1,156 (1,092-1,232; +33%)
Species diversity
Number of individuals
G
Denmark
n = 19,858; Obs. = 298
iNEXT = 340 (313-368; +12%)
iChao = 350 (332-377; +15%)
Species diversity
Number of individuals
H
Dominica
n = 288; Obs. = 34
iNEXT = 51 (20-81; +33%)
iChao = 52 (39-94; +35%)
Species diversity
Number of individuals
I
Dominican Republic
n = 1,159; Obs. = 71
iNEXT = 83 (62-104; +14%)
iChao = 85 (77-106; +17%)
Species diversity
Number of individuals
J
Ecuador
n = 15,916; Obs. = 566
iNEXT = 711 (660-762; +20%)
iChao = 743 (708-788; +24%)
Species diversity
Number of individuals
K
Egypt
n = 6,165; Obs. = 490
iNEXT = 678 (614-742; +28%)
iChao = 725 (679-782; +32%)
Species diversity
Number of individuals
L
El Salvador
n = 2,140; Obs. = 121
iNEXT = 159 (127-191; +24%)
iChao = 159 (138-204; +24%)
Species diversity
Number of individuals

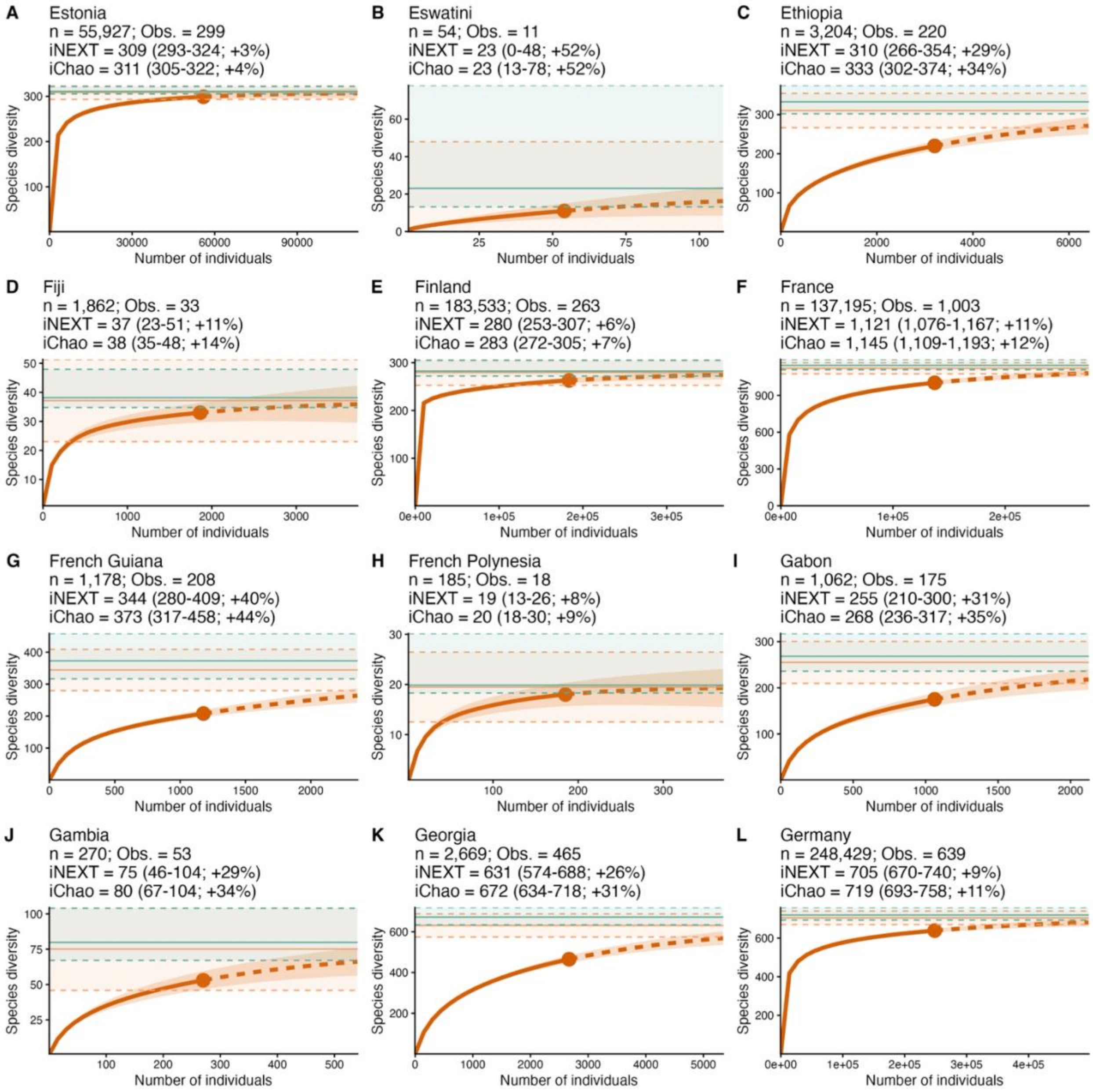
A
Estonia
n = 55,927; Obs. = 299
iNEXT = 309 (293-324; +3%)
iChao = 311 (305-322; +4%)
B
Eswatini
n = 54; Obs. = 11
iNEXT = 23 (0-48; +52%)
iChao = 23 (13-78; +52%)
C
Ethiopia
n = 3,204; Obs. = 220
iNEXT = 310 (266-354; +29%)
iChao = 333 (302-374; +34%)
D
Fiji
n = 1,862; Obs. = 33
iNEXT = 37 (23-51; +11%)
iChao = 38 (35-48; +14%)
E
Finland
n = 183,533; Obs. = 263
iNEXT = 280 (253-307; +6%)
iChao = 283 (272-305; +7%)
F
France
n = 137,195; Obs. = 1,003
iNEXT = 1,121 (1,076-1,167; +11%)
iChao = 1,145 (1,109-1,193; +12%)
G
French Guiana
n = 1,178; Obs. = 208
iNEXT = 344 (280-409; +40%)
iChao = 373 (317-458; +44%)
H
French Polynesia
n = 185; Obs. = 18
iNEXT = 19 (13-26; +8%)
iChao = 20 (18-30; +9%)
I
Gabon
n = 1,062; Obs. = 175
iNEXT = 255 (210-300; +31%)
iChao = 268 (236-317; +35%)
J
Gambia
n = 270; Obs. = 53
iNEXT = 75 (46-104; +29%)
iChao = 80 (67-104; +34%)
K
Georgia
n = 2,669; Obs. = 465
iNEXT = 631 (574-688; +26%)
iChao = 672 (634-718; +31%)
L
Germany
n = 248,429; Obs. = 639
iNEXT = 705 (670-740; +9%)
iChao = 719 (693-758; +11%)
Species diversity
Number of individuals

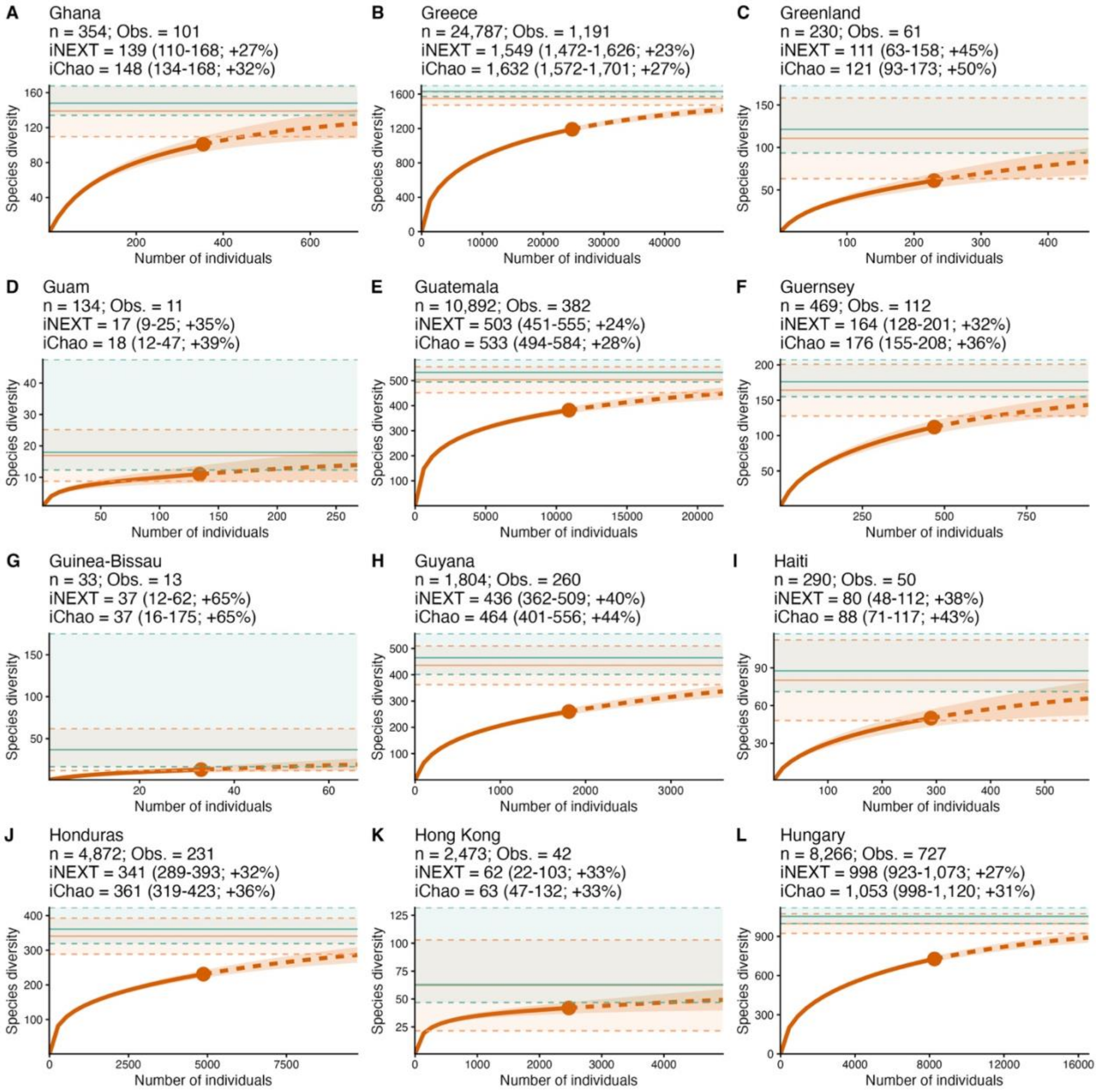
A
Ghana
n = 354; Obs. = 101
iNEXT = 139 (110-168; +27%)
iChao = 148 (134-168; +32%)
B
Greece
n = 24,787; Obs. = 1,191
iNEXT = 1,549 (1,472-1,626; +23%)
iChao = 1,632 (1,572-1,701; +27%)
C
Greenland
n = 230; Obs. = 61
iNEXT = 111 (63-158; +45%)
iChao = 121 (93-173; +50%)
D
Guam
n = 134; Obs. = 11
iNEXT = 17 (9-25; +35%)
iChao = 18 (12-47; +39%)
E
Guatemala
n = 10,892; Obs. = 382
iNEXT = 503 (451-555; +24%)
iChao = 533 (494-584; +28%)
F
Guernsey
n = 469; Obs. = 112
iNEXT = 164 (128-201; +32%)
iChao = 176 (155-208; +36%)
G
Guinea-Bissau
n = 33; Obs. = 13
iNEXT = 37 (12-62; +65%)
iChao = 37 (16-175; +65%)
H
Guyana
n = 1,804; Obs. = 260
iNEXT = 436 (362-509; +40%)
iChao = 464 (401-556; +44%)
I
Haiti
n = 290; Obs. = 50
iNEXT = 80 (48-112; +38%)
iChao = 88 (71-117; +43%)
J
Honduras
n = 4,872; Obs. = 231
iNEXT = 341 (289-393; +32%)
iChao = 361 (319-423; +36%)
K
Hong Kong
n = 2,473; Obs. = 42
iNEXT = 62 (22-103; +33%)
iChao = 63 (47-132; +33%)
L
Hungary
n = 8,266; Obs. = 727
iNEXT = 998 (923-1,073; +27%)
iChao = 1,053 (998-1,120; +31%)
Species diversity
Number of individuals

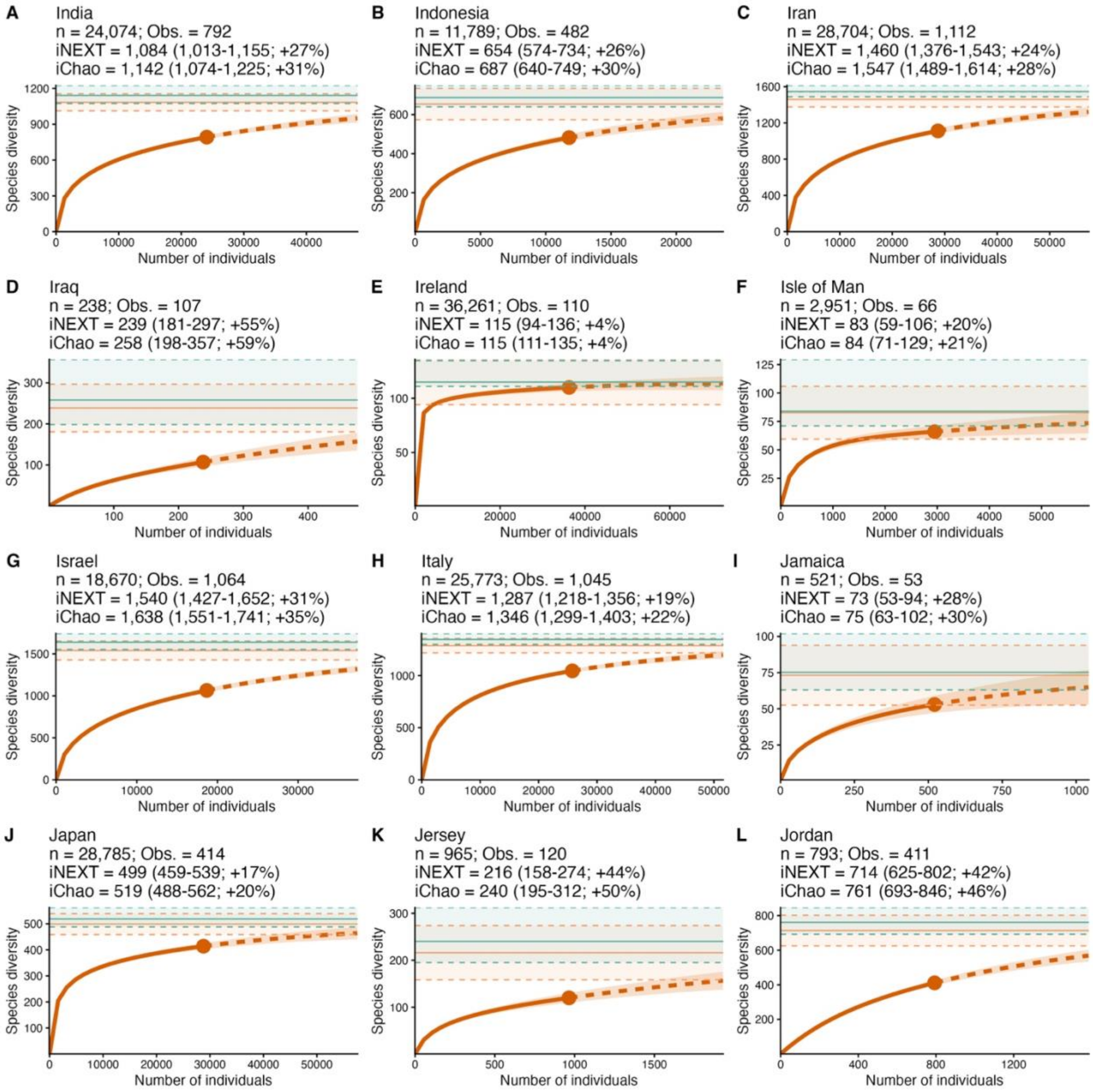
A
India
n = 24,074; Obs. = 792
iNEXT = 1,084 (1,013-1,155; +27%)
iChao = 1,142 (1,074-1,225; +31%)
B
Indonesia
n = 11,789; Obs. = 482
iNEXT = 654 (574-734; +26%)
iChao = 687 (640-749; +30%)
C
Iran
n = 28,704; Obs. = 1,112
iNEXT = 1,460 (1,376-1,543; +24%)
iChao = 1,547 (1,489-1,614; +28%)
D
Iraq
n = 238; Obs. = 107
iNEXT = 239 (181-297; +55%)
iChao = 258 (198-357; +59%)
E
Ireland
n = 36,261; Obs. = 110
iNEXT = 115 (94-136; +4%)
iChao = 115 (111-135; +4%)
F
Isle of Man
n = 2,951; Obs. = 66
iNEXT = 83 (59-106; +20%)
iChao = 84 (71-129; +21%)
G
Israel
n = 18,670; Obs. = 1,064
iNEXT = 1,540 (1,427-1,652; +31%)
iChao = 1,638 (1,551-1,741; +35%)
H
Italy
n = 25,773; Obs. = 1,045
iNEXT = 1,287 (1,218-1,356; +19%)
iChao = 1,346 (1,299-1,403; +22%)
I
Jamaica
n = 521; Obs. = 53
iNEXT = 73 (53-94; +28%)
iChao = 75 (63-102; +30%)
J
Japan
n = 28,785; Obs. = 414
iNEXT = 499 (459-539; +17%)
iChao = 519 (488-562; +20%)
K
Jersey
n = 965; Obs. = 120
iNEXT = 216 (158-274; +44%)
iChao = 240 (195-312; +50%)
L
Jordan
n = 793; Obs. = 411
iNEXT = 714 (625-802; +42%)
iChao = 761 (693-846; +46%)
Species diversity
Number of individuals

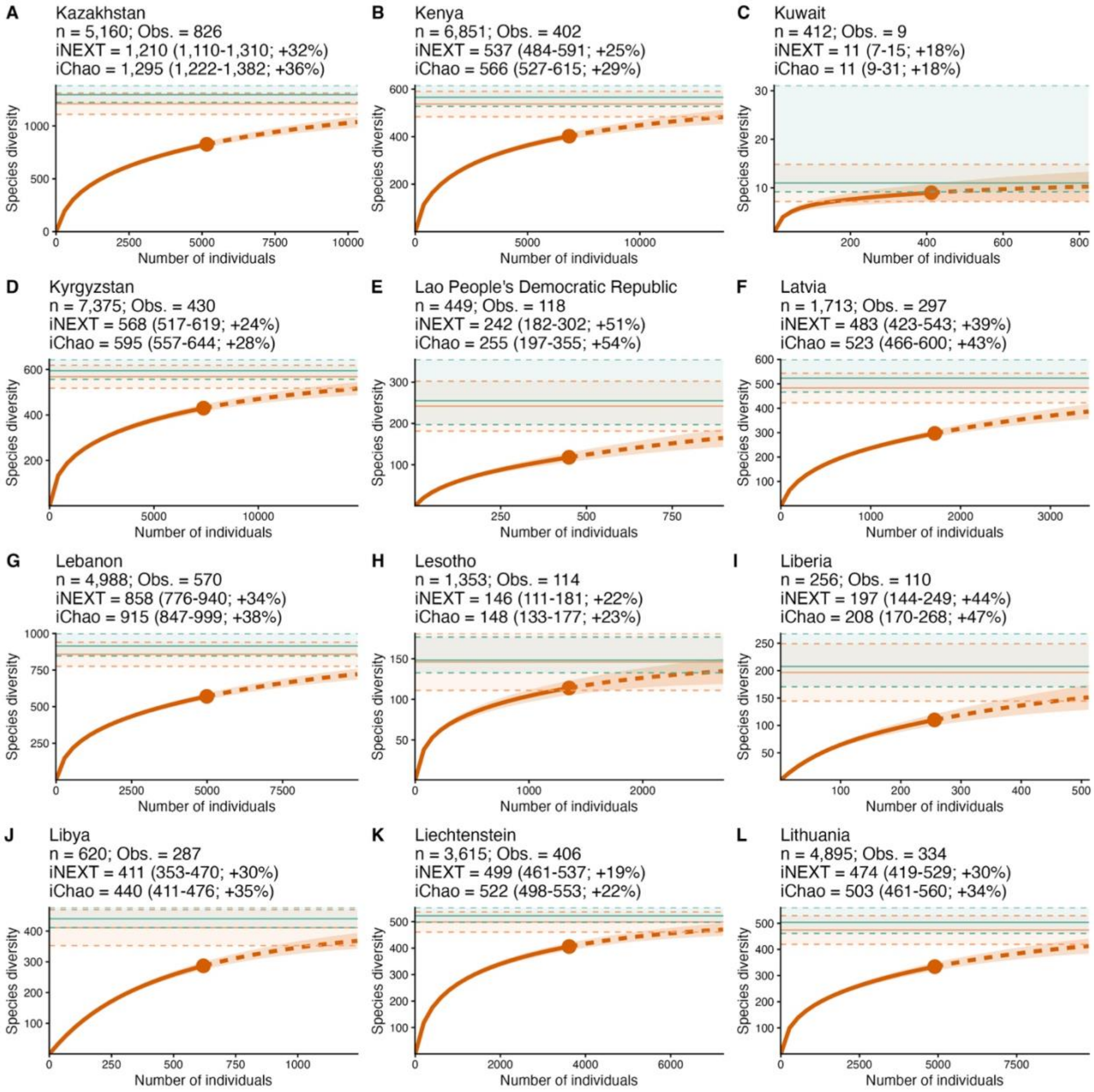
A
Kazakhstan
n = 5,160; Obs. = 826
iNEXT = 1,210 (1,110-1,310; +32%)
iChao = 1,295 (1,222-1,382; +36%)
B
Kenya
n = 6,851; Obs. = 402
iNEXT = 537 (484-591; +25%)
iChao = 566 (527-615; +29%)
C
Kuwait
n = 412; Obs. = 9
iNEXT = 11 (7-15; +18%)
iChao = 11 (9-31; +18%)
D
Kyrgyzstan
n = 7,375; Obs. = 430
iNEXT = 568 (517-619; +24%)
iChao = 595 (557-644; +28%)
E
Lao People's Democratic Republic
n = 449; Obs. = 118
iNEXT = 242 (182-302; +51%)
iChao = 255 (197-355; +54%)
F
Latvia
n = 1,713; Obs. = 297
iNEXT = 483 (423-543; +39%)
iChao = 523 (466-600; +43%)
G
Lebanon
n = 4,988; Obs. = 570
iNEXT = 858 (776-940; +34%)
iChao = 915 (847-999; +38%)
H
Lesotho
n = 1,353; Obs. = 114
iNEXT = 146 (111-181; +22%)
iChao = 148 (133-177; +23%)
I
Liberia
n = 256; Obs. = 110
iNEXT = 197 (144-249; +44%)
iChao = 208 (170-268; +47%)
J
Libya
n = 620; Obs. = 287
iNEXT = 411 (353-470; +30%)
iChao = 440 (411-476; +35%)
K
Liechtenstein
n = 3,615; Obs. = 406
iNEXT = 499 (461-537; +19%)
iChao = 522 (498-553; +22%)
L
Lithuania
n = 4,895; Obs. = 334
iNEXT = 474 (419-529; +30%)
iChao = 503 (461-560; +34%)
Species diversity
Number of individuals

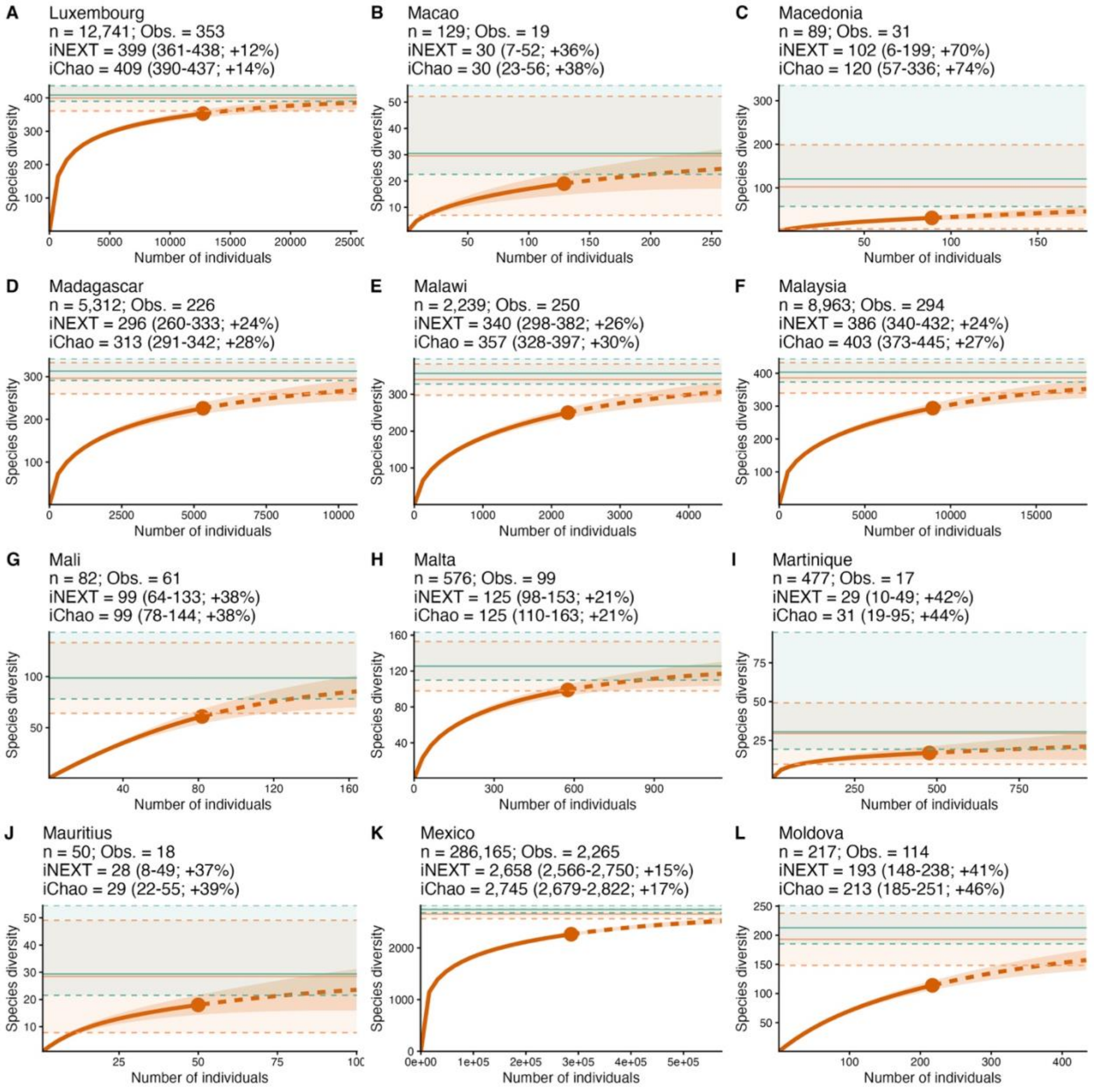
A
Luxembourg
n = 12,741; Obs. = 353
iNEXT = 399 (361-438; +12%)
iChao = 409 (390-437; +14%)
B
Macao
n = 129; Obs. = 19
iNEXT = 30 (7-52; +36%)
iChao = 30 (23-56; +38%)
C
Macedonia
n = 89; Obs. = 31
iNEXT = 102 (6-199; +70%)
iChao = 120 (57-336; +74%)
D
Madagascar
n = 5,312; Obs. = 226
iNEXT = 296 (260-333; +24%)
iChao = 313 (291-342; +28%)
E
Malawi
n = 2,239; Obs. = 250
iNEXT = 340 (298-382; +26%)
iChao = 357 (328-397; +30%)
F
Malaysia
n = 8,963; Obs. = 294
iNEXT = 386 (340-432; +24%)
iChao = 403 (373-445; +27%)
G
Mali
n = 82; Obs. = 61
iNEXT = 99 (64-133; +38%)
iChao = 99 (78-144; +38%)
H
Malta
n = 576; Obs. = 99
iNEXT = 125 (98-153; +21%)
iChao = 125 (110-163; +21%)
I
Martinique
n = 477; Obs. = 17
iNEXT = 29 (10-49; +42%)
iChao = 31 (19-95; +44%)
J
Mauritius
n = 50; Obs. = 18
iNEXT = 28 (8-49; +37%)
iChao = 29 (22-55; +39%)
K
Mexico
n = 286,165; Obs. = 2,265
iNEXT = 2,658 (2,566-2,750; +15%)
iChao = 2,745 (2,679-2,822; +17%)
L
Moldova
n = 217; Obs. = 114
iNEXT = 193 (148-238; +41%)
iChao = 213 (185-251; +46%)
Species diversity
Number of individuals

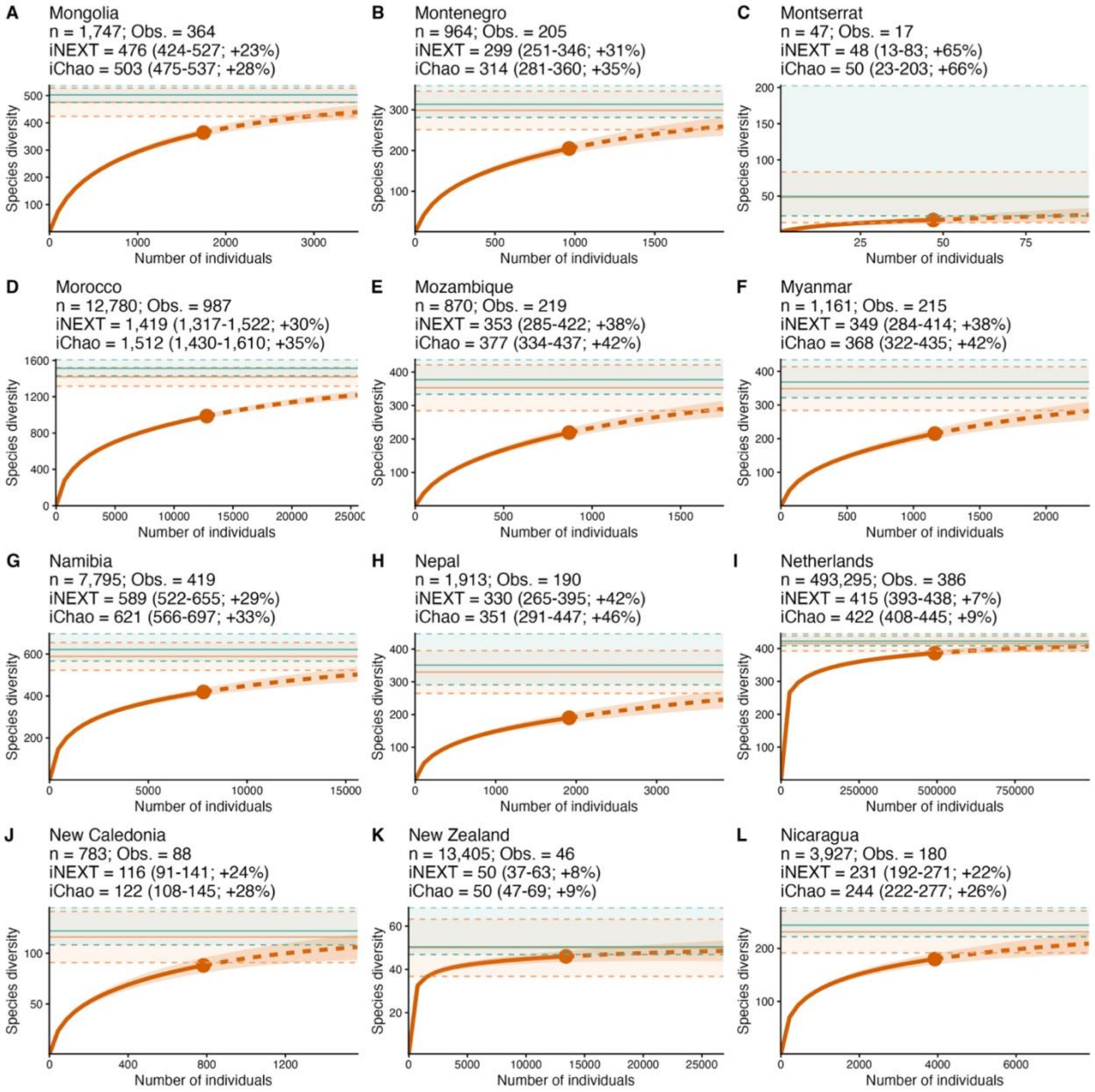
A
Mongolia
n = 1,747; Obs. = 364
iNEXT = 476 (424-527; +23%)
iChao = 503 (475-537; +28%)
Species diversity
Number of individuals
B
Montenegro
n = 964; Obs. = 205
iNEXT = 299 (251-346; +31%)
iChao = 314 (281-360; +35%)
Species diversity
Number of individuals
C
Montserrat
n = 47; Obs. = 17
iNEXT = 48 (13-83; +65%)
iChao = 50 (23-203; +66%)
Species diversity
Number of individuals
D
Morocco
n = 12,780; Obs. = 987
iNEXT = 1,419 (1,317-1,522; +30%)
iChao = 1,512 (1,430-1,610; +35%)
Species diversity
Number of individuals
E
Mozambique
n = 870; Obs. = 219
iNEXT = 353 (285-422; +38%)
iChao = 377 (334-437; +42%)
Species diversity
Number of individuals
F
Myanmar
n = 1,161; Obs. = 215
iNEXT = 349 (284-414; +38%)
iChao = 368 (322-435; +42%)
Species diversity
Number of individuals
G
Namibia
n = 7,795; Obs. = 419
iNEXT = 589 (522-655; +29%)
iChao = 621 (566-697; +33%)
Species diversity
Number of individuals
H
Nepal
n = 1,913; Obs. = 190
iNEXT = 330 (265-395; +42%)
iChao = 351 (291-447; +46%)
Species diversity
Number of individuals
I
Netherlands
n = 493,295; Obs. = 386
iNEXT = 415 (393-438; +7%)
iChao = 422 (408-445; +9%)
Species diversity
Number of individuals
J
New Caledonia
n = 783; Obs. = 88
iNEXT = 116 (91-141; +24%)
iChao = 122 (108-145; +28%)
Species diversity
Number of individuals
K
New Zealand
n = 13,405; Obs. = 46
iNEXT = 50 (37-63; +8%)
iChao = 50 (47-69; +9%)
Species diversity
Number of individuals
L
Nicaragua
n = 3,927; Obs. = 180
iNEXT = 231 (192-271; +22%)
iChao = 244 (222-277; +26%)
Species diversity
Number of individuals

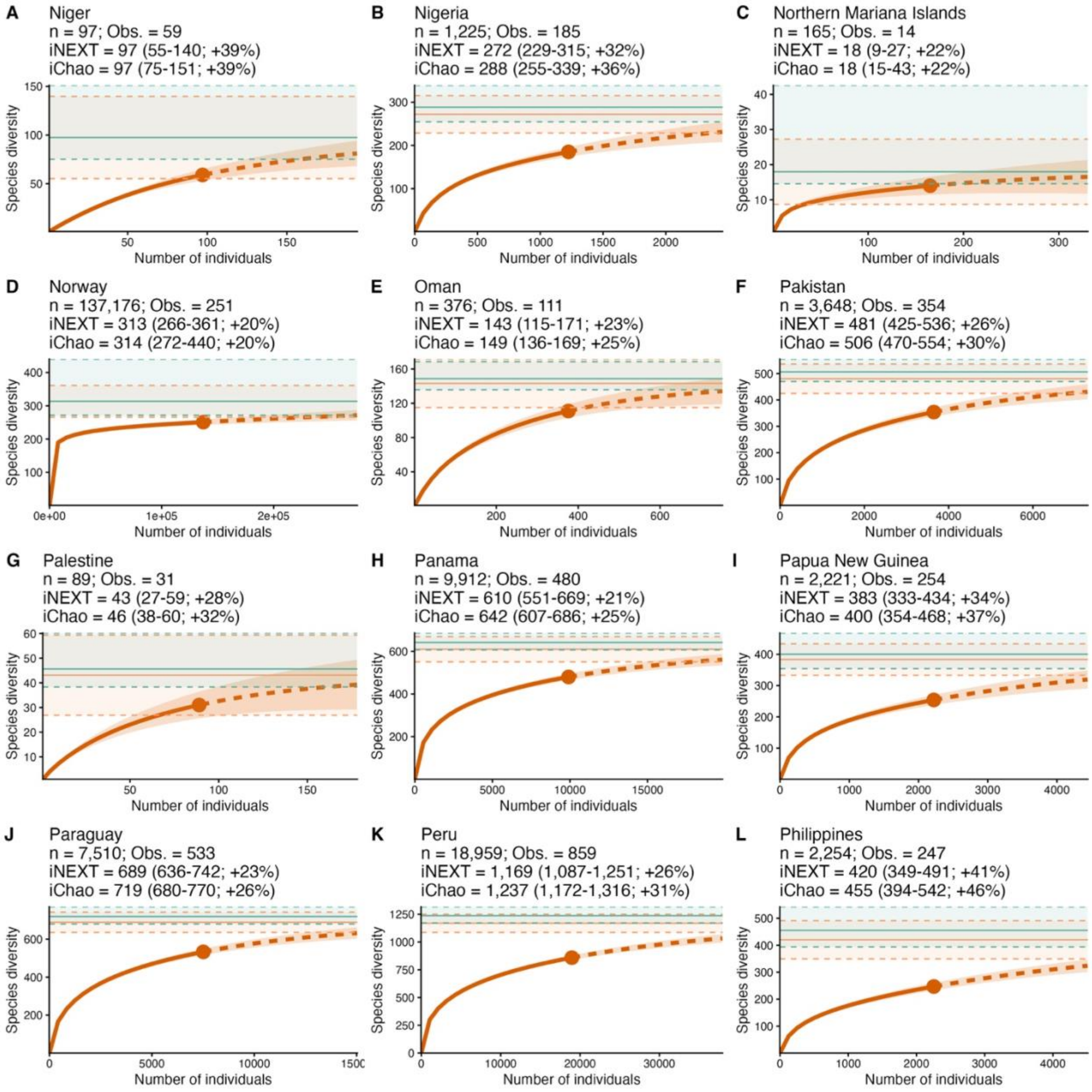
A
Niger
n = 97; Obs. = 59
iNEXT = 97 (55-140; +39%)
iChao = 97 (75-151; +39%)
B
Nigeria
n = 1,225; Obs. = 185
iNEXT = 272 (229-315; +32%)
iChao = 288 (255-339; +36%)
C
Northern Mariana Islands
n = 165; Obs. = 14
iNEXT = 18 (9-27; +22%)
iChao = 18 (15-43; +22%)
D
Norway
n = 137,176; Obs. = 251
iNEXT = 313 (266-361; +20%)
iChao = 314 (272-440; +20%)
E
Oman
n = 376; Obs. = 111
iNEXT = 143 (115-171; +23%)
iChao = 149 (136-169; +25%)
F
Pakistan
n = 3,648; Obs. = 354
iNEXT = 481 (425-536; +26%)
iChao = 506 (470-554; +30%)
G
Palestine
n = 89; Obs. = 31
iNEXT = 43 (27-59; +28%)
iChao = 46 (38-60; +32%)
H
Panama
n = 9,912; Obs. = 480
iNEXT = 610 (551-669; +21%)
iChao = 642 (607-686; +25%)
I
Papua New Guinea
n = 2,221; Obs. = 254
iNEXT = 383 (333-434; +34%)
iChao = 400 (354-468; +37%)
J
Paraguay
n = 7,510; Obs. = 533
iNEXT = 689 (636-742; +23%)
iChao = 719 (680-770; +26%)
K
Peru
n = 18,959; Obs. = 859
iNEXT = 1,169 (1,087-1,251; +26%)
iChao = 1,237 (1,172-1,316; +31%)
L
Philippines
n = 2,254; Obs. = 247
iNEXT = 420 (349-491; +41%)
iChao = 455 (394-542; +46%)
Species diversity
Number of individuals

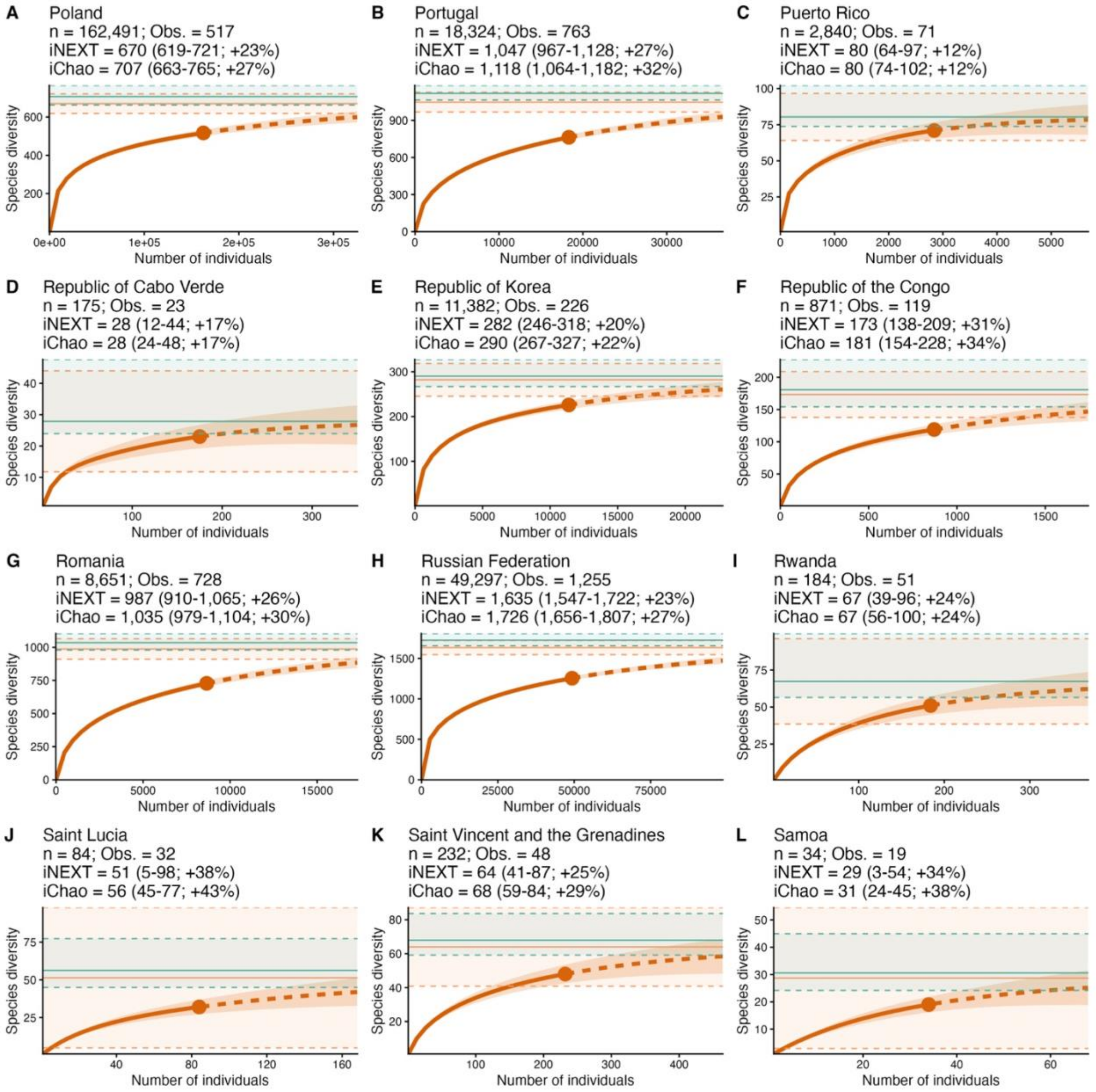
A
Poland
n = 162,491; Obs. = 517
iNEXT = 670 (619-721; +23%)
iChao = 707 (663-765; +27%)
B
Portugal
n = 18,324; Obs. = 763
iNEXT = 1,047 (967-1,128; +27%)
iChao = 1,118 (1,064-1,182; +32%)
C
Puerto Rico
n = 2,840; Obs. = 71
iNEXT = 80 (64-97; +12%)
iChao = 80 (74-102; +12%)
D
Republic of Cabo Verde
n = 175; Obs. = 23
iNEXT = 28 (12-44; +17%)
iChao = 28 (24-48; +17%)
E
Republic of Korea
n = 11,382; Obs. = 226
iNEXT = 282 (246-318; +20%)
iChao = 290 (267-327; +22%)
F
Republic of the Congo
n = 871; Obs. = 119
iNEXT = 173 (138-209; +31%)
iChao = 181 (154-228; +34%)
G
Romania
n = 8,651; Obs. = 728
iNEXT = 987 (910-1,065; +26%)
iChao = 1,035 (979-1,104; +30%)
H
Russian Federation
n = 49,297; Obs. = 1,255
iNEXT = 1,635 (1,547-1,722; +23%)
iChao = 1,726 (1,656-1,807; +27%)
I
Rwanda
n = 184; Obs. = 51
iNEXT = 67 (39-96; +24%)
iChao = 67 (56-100; +24%)
J
Saint Lucia
n = 84; Obs. = 32
iNEXT = 51 (5-98; +38%)
iChao = 56 (45-77; +43%)
K
Saint Vincent and the Grenadines
n = 232; Obs. = 48
iNEXT = 64 (41-87; +25%)
iChao = 68 (59-84; +29%)
L
Samoa
n = 34; Obs. = 19
iNEXT = 29 (3-54; +34%)
iChao = 31 (24-45; +38%)
Species diversity
Number of individuals

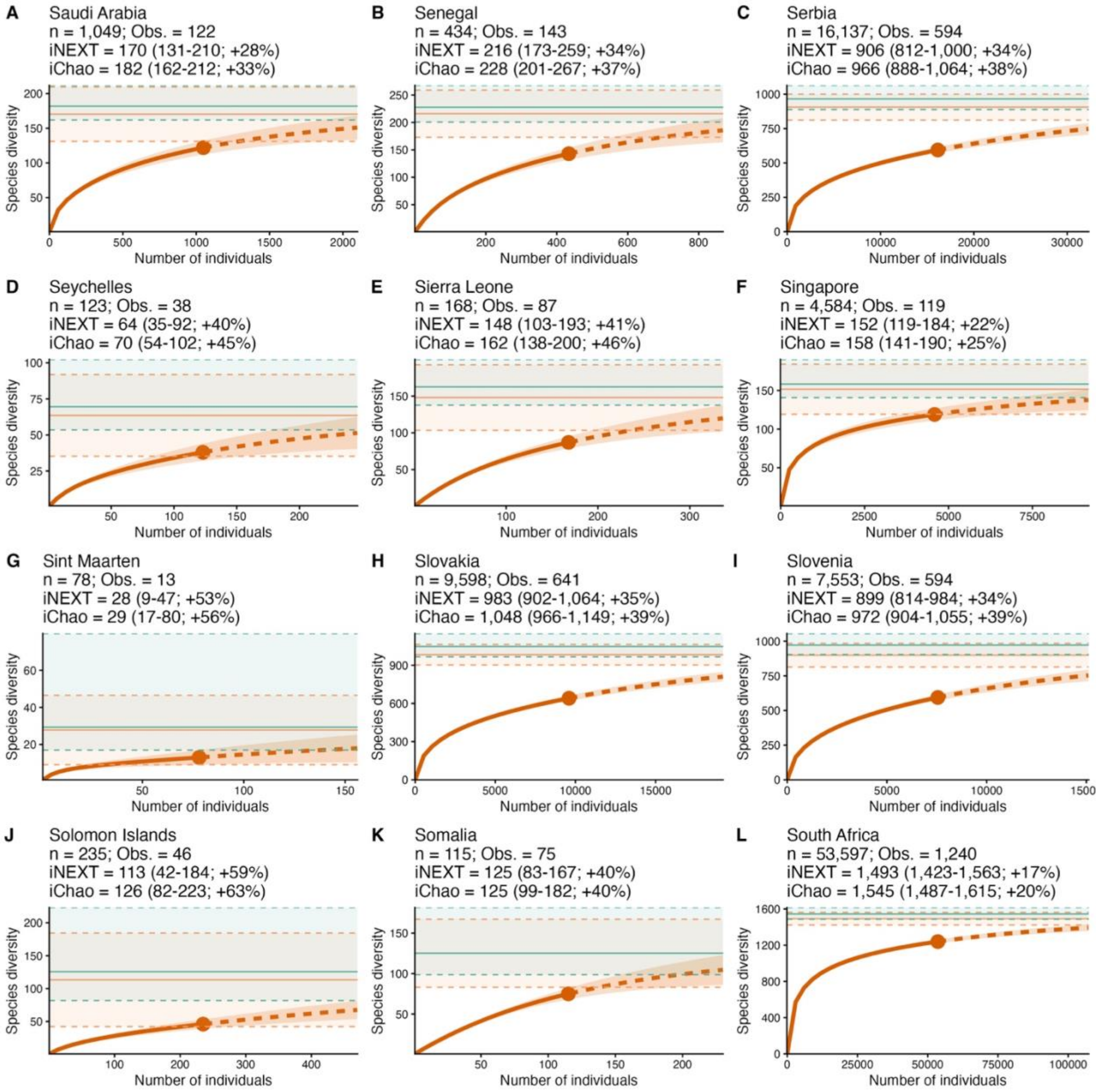
A
Saudi Arabia
n = 1,049; Obs. = 122
iNEXT = 170 (131-210; +28%)
iChao = 182 (162-212; +33%)
B
Senegal
n = 434; Obs. = 143
iNEXT = 216 (173-259; +34%)
iChao = 228 (201-267; +37%)
C
Serbia
n = 16,137; Obs. = 594
iNEXT = 906 (812-1,000; +34%)
iChao = 966 (888-1,064; +38%)
D
Seychelles
n = 123; Obs. = 38
iNEXT = 64 (35-92; +40%)
iChao = 70 (54-102; +45%)
E
Sierra Leone
n = 168; Obs. = 87
iNEXT = 148 (103-193; +41%)
iChao = 162 (138-200; +46%)
F
Singapore
n = 4,584; Obs. = 119
iNEXT = 152 (119-184; +22%)
iChao = 158 (141-190; +25%)
G
Sint Maarten
n = 78; Obs. = 13
iNEXT = 28 (9-47; +53%)
iChao = 29 (17-80; +56%)
H
Slovakia
n = 9,598; Obs. = 641
iNEXT = 983 (902-1,064; +35%)
iChao = 1,048 (966-1,149; +39%)
I
Slovenia
n = 7,553; Obs. = 594
iNEXT = 899 (814-984; +34%)
iChao = 972 (904-1,055; +39%)
J
Solomon Islands
n = 235; Obs. = 46
iNEXT = 113 (42-184; +59%)
iChao = 126 (82-223; +63%)
K
Somalia
n = 115; Obs. = 75
iNEXT = 125 (83-167; +40%)
iChao = 125 (99-182; +40%)
L
South Africa
n = 53,597; Obs. = 1,240
iNEXT = 1,493 (1,423-1,563; +17%)
iChao = 1,545 (1,487-1,615; +20%)
Species diversity
Number of individuals

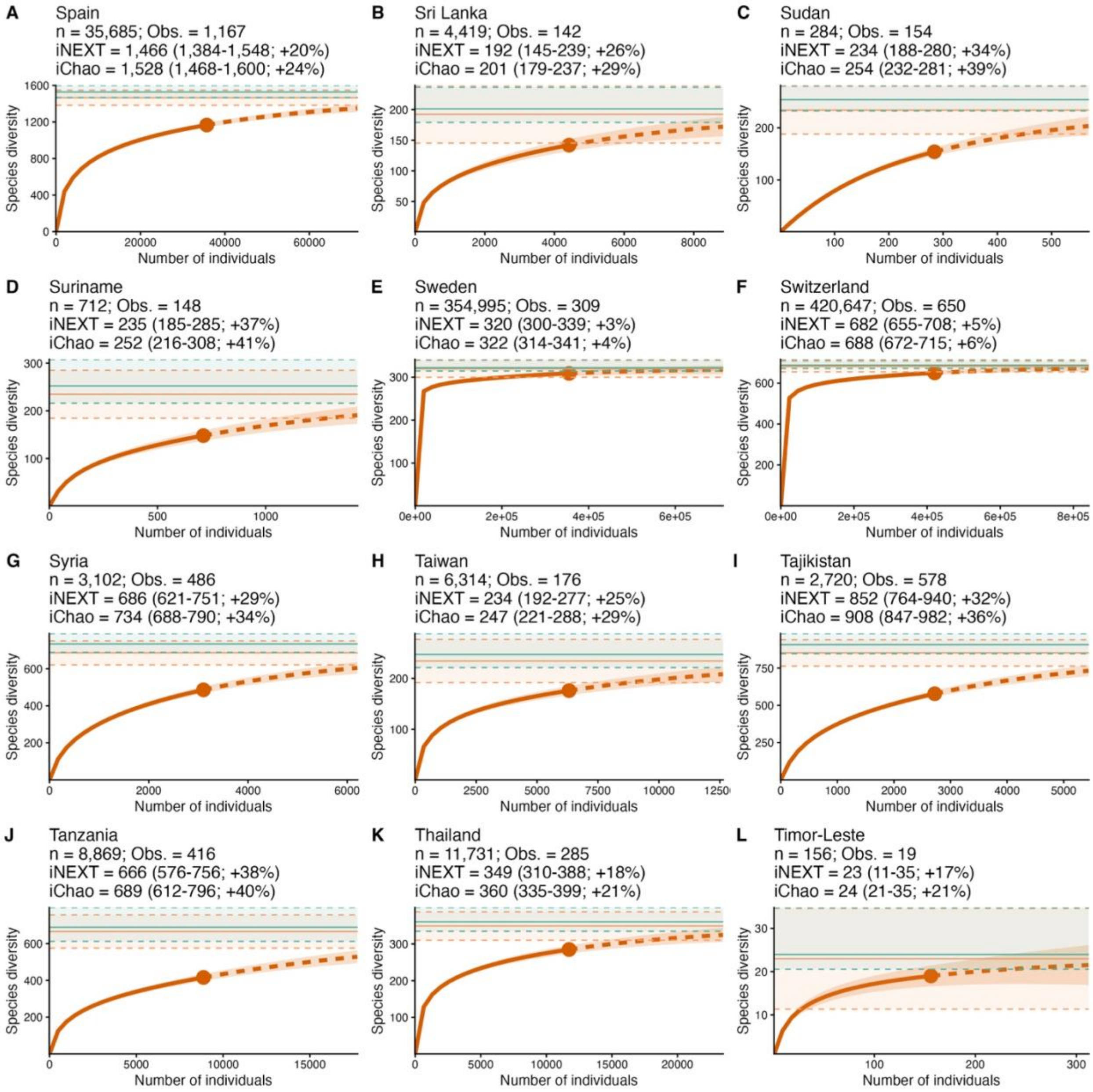
A
Spain
n = 35,685; Obs. = 1,167
iNEXT = 1,466 (1,384-1,548; +20%)
iChao = 1,528 (1,468-1,600; +24%)
B
Sri Lanka
n = 4,419; Obs. = 142
iNEXT = 192 (145-239; +26%)
iChao = 201 (179-237; +29%)
C
Sudan
n = 284; Obs. = 154
iNEXT = 234 (188-280; +34%)
iChao = 254 (232-281; +39%)
D
Suriname
n = 712; Obs. = 148
iNEXT = 235 (185-285; +37%)
iChao = 252 (216-308; +41%)
E
Sweden
n = 354,995; Obs. = 309
iNEXT = 320 (300-339; +3%)
iChao = 322 (314-341; +4%)
F
Switzerland
n = 420,647; Obs. = 650
iNEXT = 682 (655-708; +5%)
iChao = 688 (672-715; +6%)
G
Syria
n = 3,102; Obs. = 486
iNEXT = 686 (621-751; +29%)
iChao = 734 (688-790; +34%)
H
Taiwan
n = 6,314; Obs. = 176
iNEXT = 234 (192-277; +25%)
iChao = 247 (221-288; +29%)
I
Tajikistan
n = 2,720; Obs. = 578
iNEXT = 852 (764-940; +32%)
iChao = 908 (847-982; +36%)
J
Tanzania
n = 8,869; Obs. = 416
iNEXT = 666 (576-756; +38%)
iChao = 689 (612-796; +40%)
K
Thailand
n = 11,731; Obs. = 285
iNEXT = 349 (310-388; +18%)
iChao = 360 (335-399; +21%)
L
Timor-Leste
n = 156; Obs. = 19
iNEXT = 23 (11-35; +17%)
iChao = 24 (21-35; +21%)
Species diversity
Number of individuals

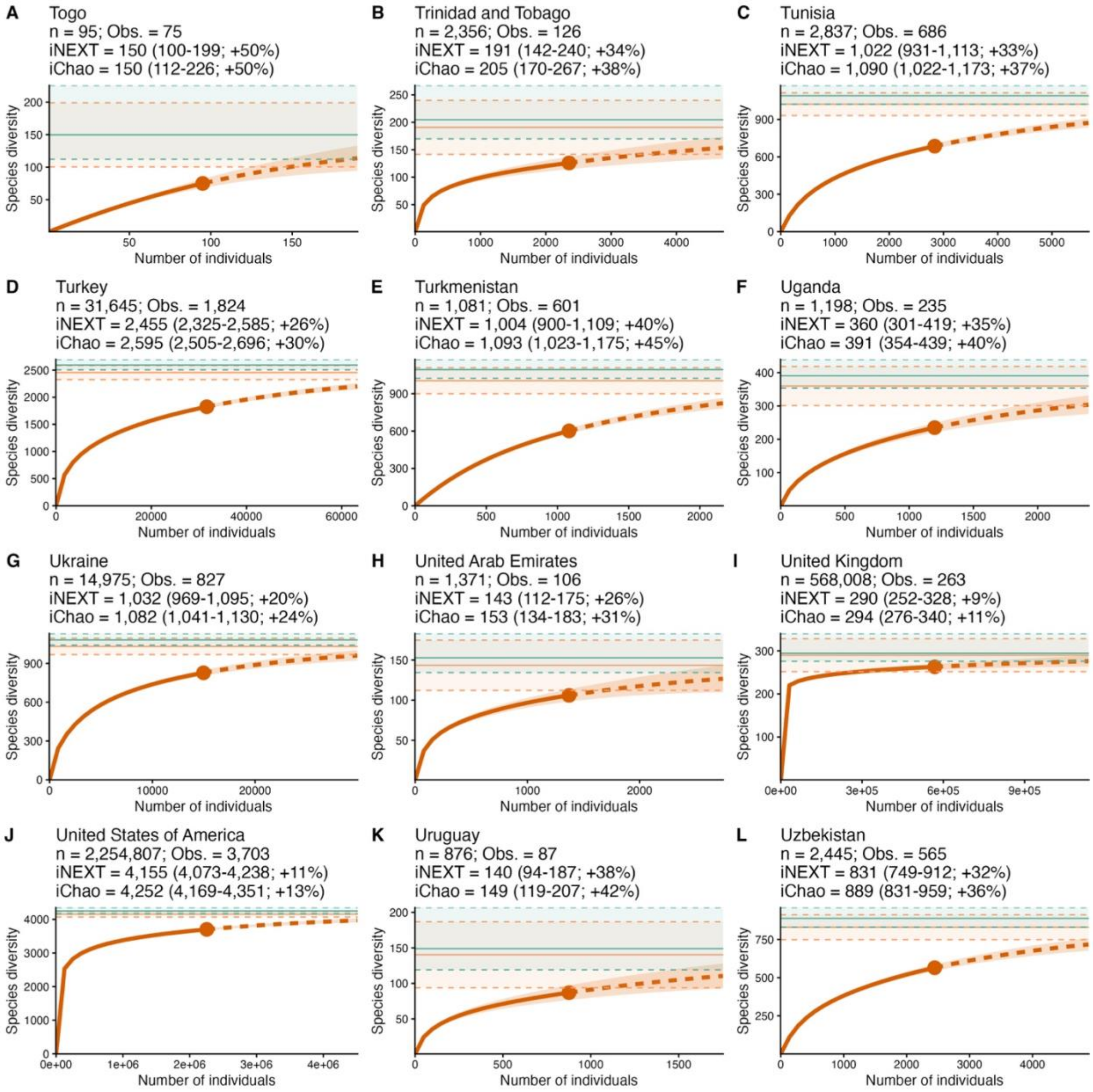
A
Togo
n = 95; Obs. = 75
iNEXT = 150 (100-199; +50%)
iChao = 150 (112-226; +50%)
B
Trinidad and Tobago
n = 2,356; Obs. = 126
iNEXT = 191 (142-240; +34%)
iChao = 205 (170-267; +38%)
C
Tunisia
n = 2,837; Obs. = 686
iNEXT = 1,022 (931-1,113; +33%)
iChao = 1,090 (1,022-1,173; +37%)
D
Turkey
n = 31,645; Obs. = 1,824
iNEXT = 2,455 (2,325-2,585; +26%)
iChao = 2,595 (2,505-2,696; +30%)
E
Turkmenistan
n = 1,081; Obs. = 601
iNEXT = 1,004 (900-1,109; +40%)
iChao = 1,093 (1,023-1,175; +45%)
F
Uganda
n = 1,198; Obs. = 235
iNEXT = 360 (301-419; +35%)
iChao = 391 (354-439; +40%)
G
Ukraine
n = 14,975; Obs. = 827
iNEXT = 1,032 (969-1,095; +20%)
iChao = 1,082 (1,041-1,130; +24%)
H
United Arab Emirates
n = 1,371; Obs. = 106
iNEXT = 143 (112-175; +26%)
iChao = 153 (134-183; +31%)
I
United Kingdom
n = 568,008; Obs. = 263
iNEXT = 290 (252-328; +9%)
iChao = 294 (276-340; +11%)
J
United States of America
n = 2,254,807; Obs. = 3,703
iNEXT = 4,155 (4,073-4,238; +11%)
iChao = 4,252 (4,169-4,351; +13%)
K
Uruguay
n = 876; Obs. = 87
iNEXT = 140 (94-187; +38%)
iChao = 149 (119-207; +42%)
L
Uzbekistan
n = 2,445; Obs. = 565
iNEXT = 831 (749-912; +32%)
iChao = 889 (831-959; +36%)
Species diversity
Number of individuals

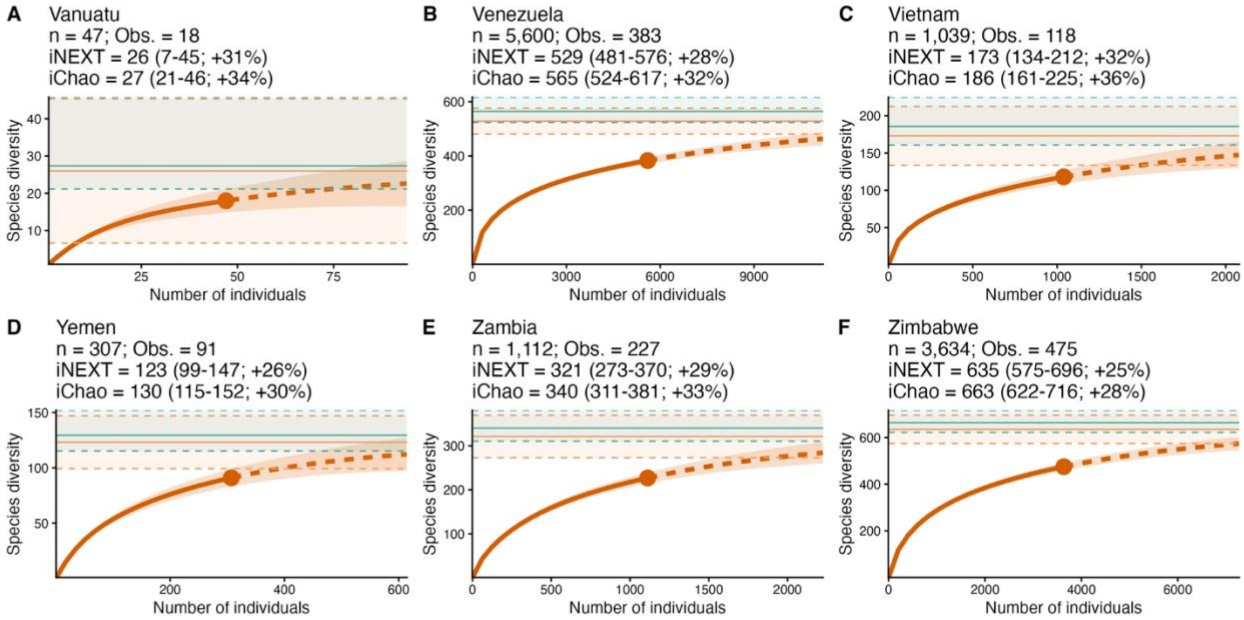

Fig. S4.

The country plots showing the estimated species accumulation curves from iNEXT (orange curve), the estimated value and 95% confidence intervals from iNEXT (orange) and iChao (green) over the number of individuals. The dashed curve indicates the iNEXT extrapolation past 100% of the empirical sample size. Estimates are from a single sample of the literature curve combined with the empirical data. Estimates are from a single sample of the literature curve combined with the empirical data. The original publication and original input data can be downloaded from https://doi.org/10.25451/flinders.21709757. Source data are provided as a Source Data file.

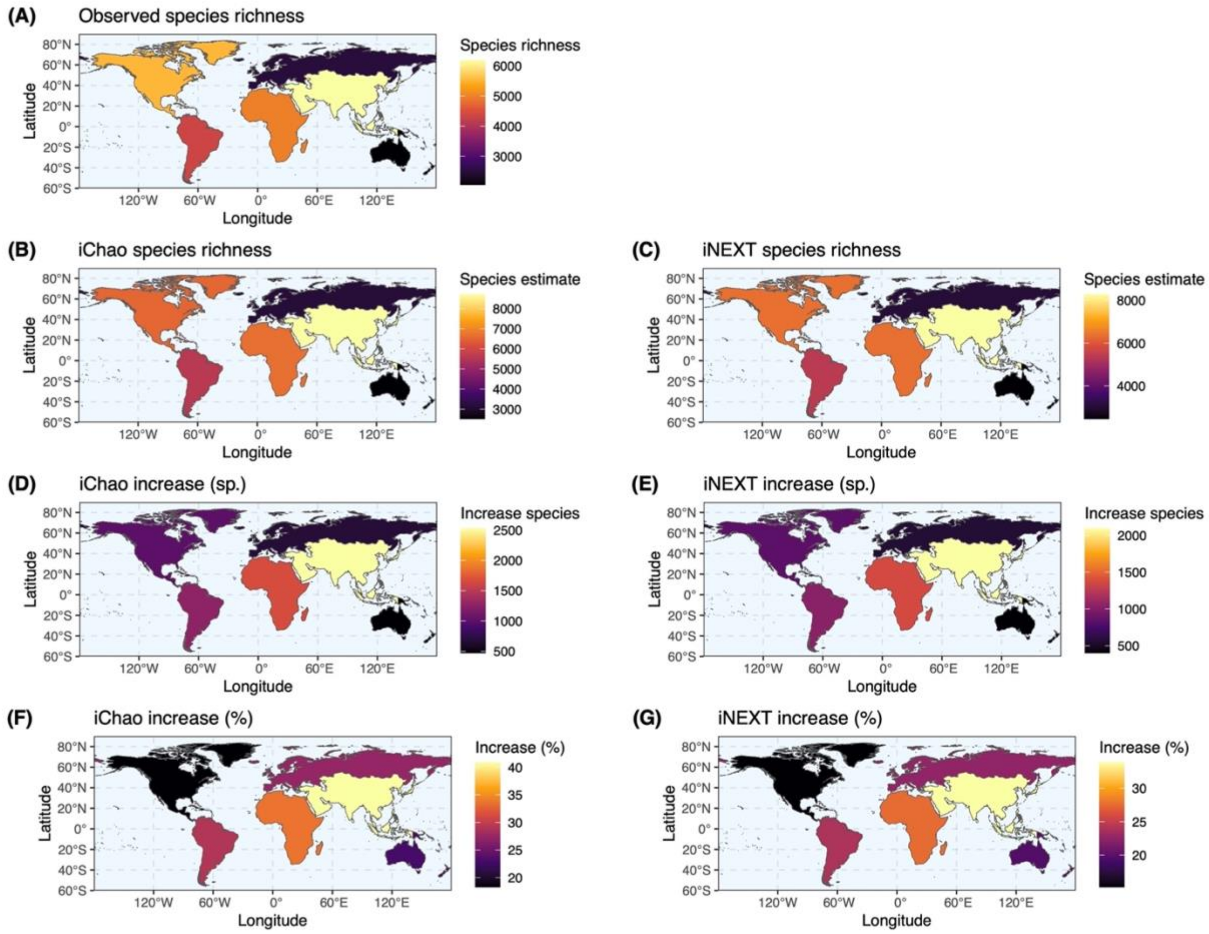

Fig. S5.

Continent-scale maps showing (A) observed species richness; species richness estimated using (B) iChao and (C) iNEXT; the estimated increase in species richness estimated using (D) iChao and (E) iNEXT; and the estimated percentage increase estimated using (F) iChao and (G) iNEXT. Estimates are from the median values of 100 iterations sampling the literature curve combined with the empirical data. The original publication and original input data can be downloaded from https://doi.org/10.25451/flinders.21709757. Source data are provided as a Source Data file. Base maps were sourced using rnaturalearth version 1.0.1 and rnaturalearthdata version 1.0.0.

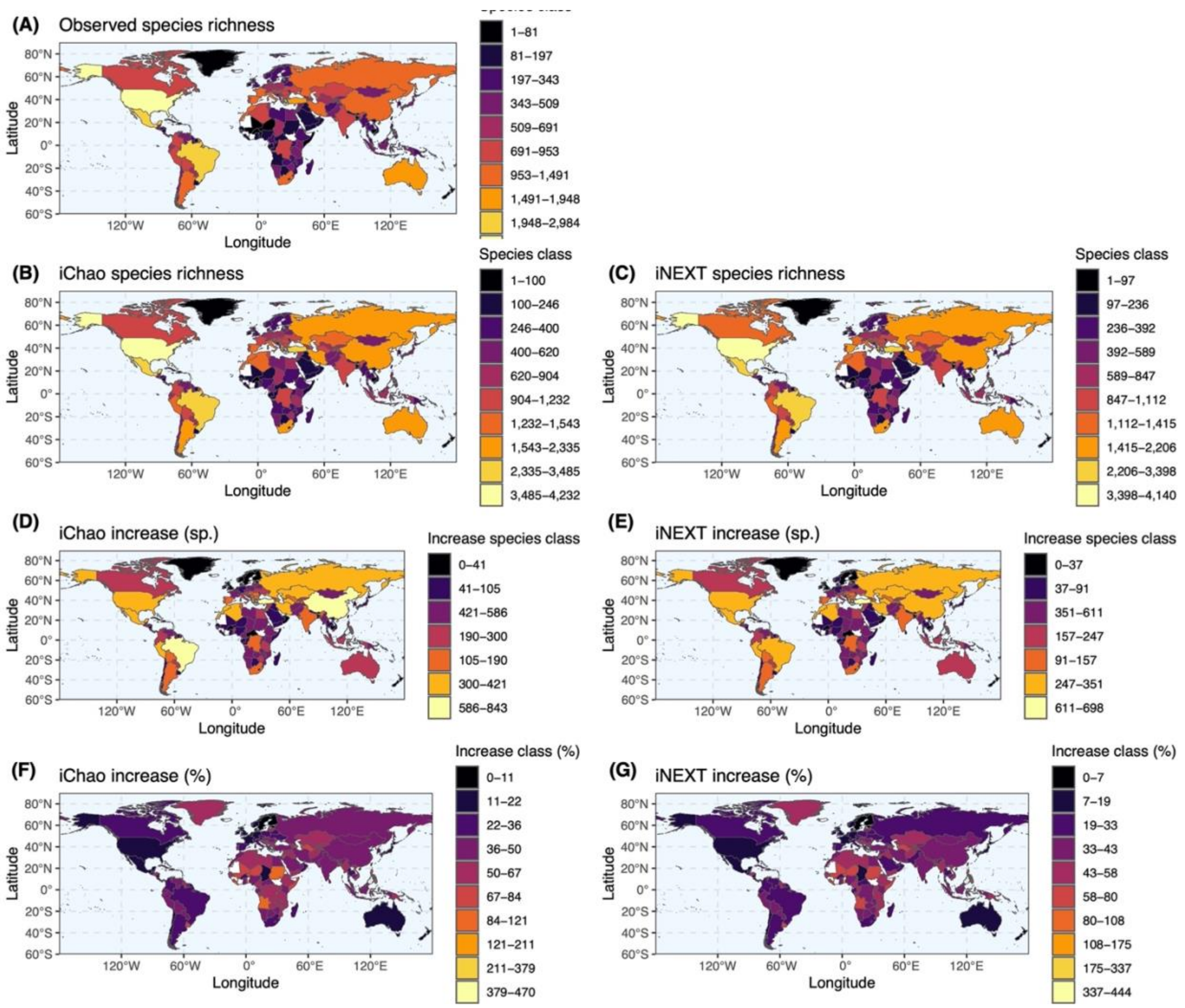

Fig. S6.

Country-scale maps showing (A) observed species richness; species richness estimated using (B) iChao and (C) iNEXT; the estimated increase in species richness estimated using (D) iChao and (E) iNEXT; and the estimated percentage increase estimated using (F) iChao and (G) iNEXT. Estimates are from the median values of 100 iterations sampling the literature curve combined with the empirical data. The original publication and original input data can be downloaded from https://doi.org/10.25451/flinders.21709757. Source data are provided as a Source Data file. Base maps were sourced using rnaturalearth version 1.0.1 and rnaturalearthdata version 1.0.0.

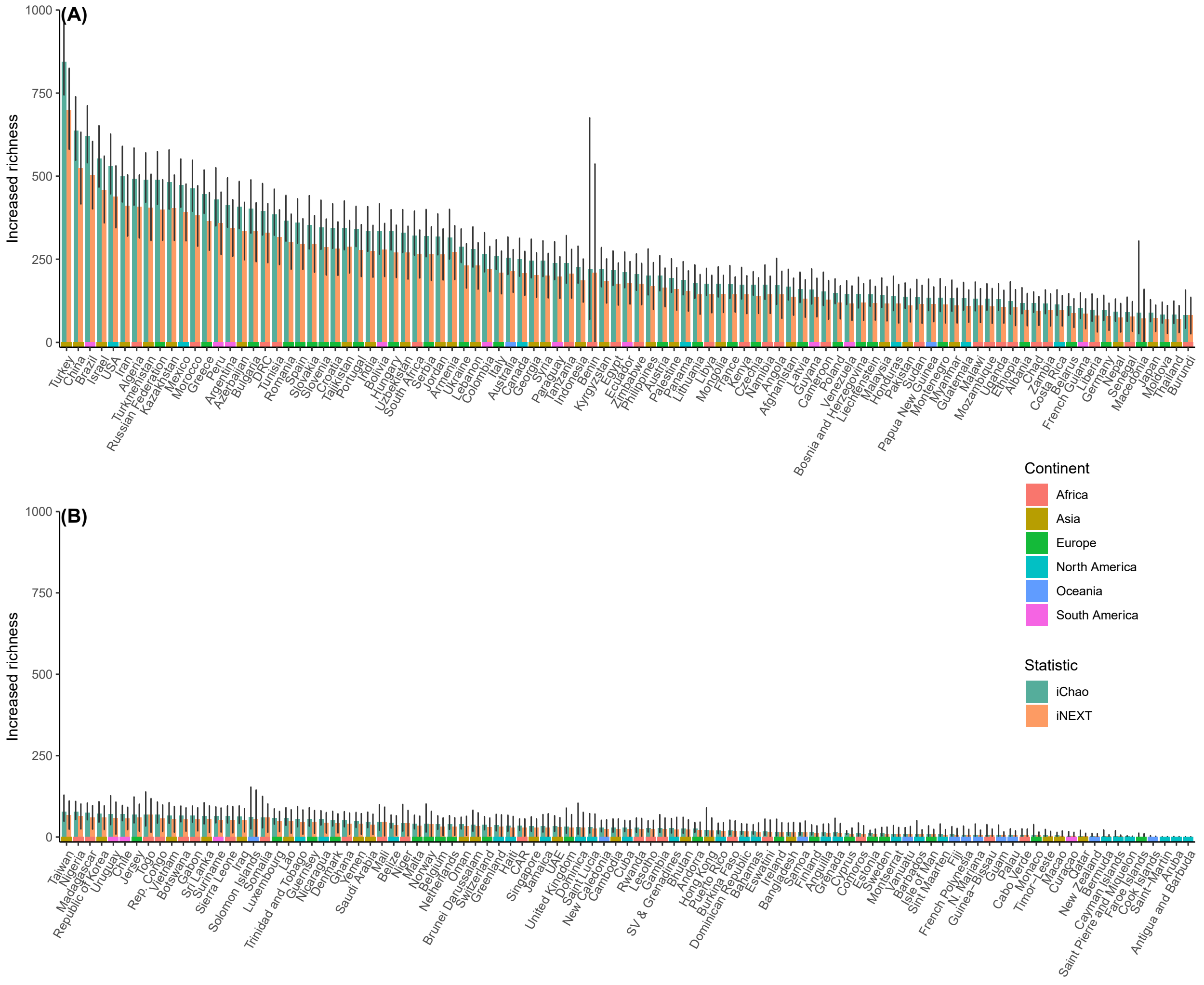

Fig. S7.

Bar plots showing the top (A) and bottom (B) half countries for increased species. Bars show numbers estimated using the statistics iChao (green) and iNEXT (orange). Lower bars indicate continent. Estimates are the median values from 100 samples of the literature curve and bars indicate 95% confidence intervals. The original publication and original input data can be downloaded from https://doi.org/10.25451/flinders.21709757. Source data are provided as a Source Data file.

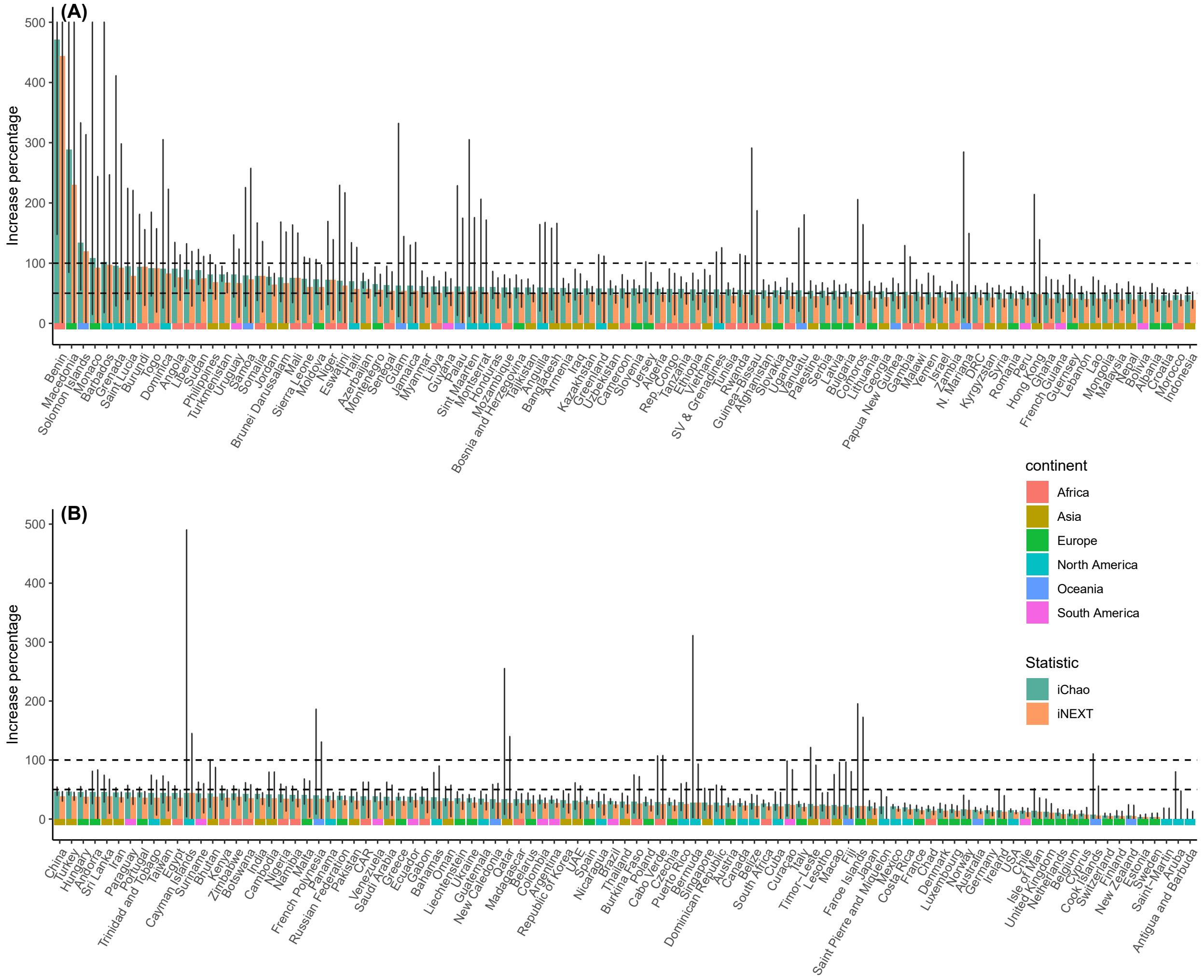

Fig. S8.

Bar plots showing the top (A) and bottom (B) half countries for increased species percentage. Note that the Y-axis is cut at 500% and that the horizontal dashed bars show 100% and 50% increases. Bars show numbers estimated using the statistics iChao (green) and iNEXT (orange). Lower bars indicate continent. Estimates are the median values from 100 samples of the literature curve and bars indicate 95% confidence intervals. The original publication and original input data can be downloaded from https://doi.org/10.25451/flinders.21709757. Source data are provided as a Source Data file.

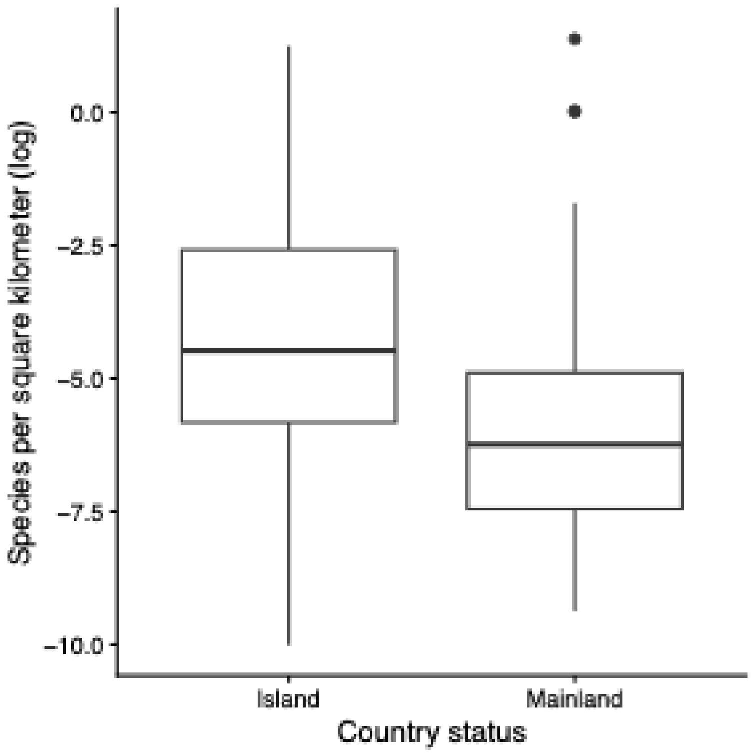

Fig. S9.

Box plot showing the log of species per square kilometer for countries that are islands or mainland. A two-sided Wilcoxon rank sum test found a significant difference between the groups ($p = 5.0x10^{-8}$). The original publication and original input data can be downloaded from https://doi.org/10.25451/flinders.21709757. Source data are provided as a Source Data file.

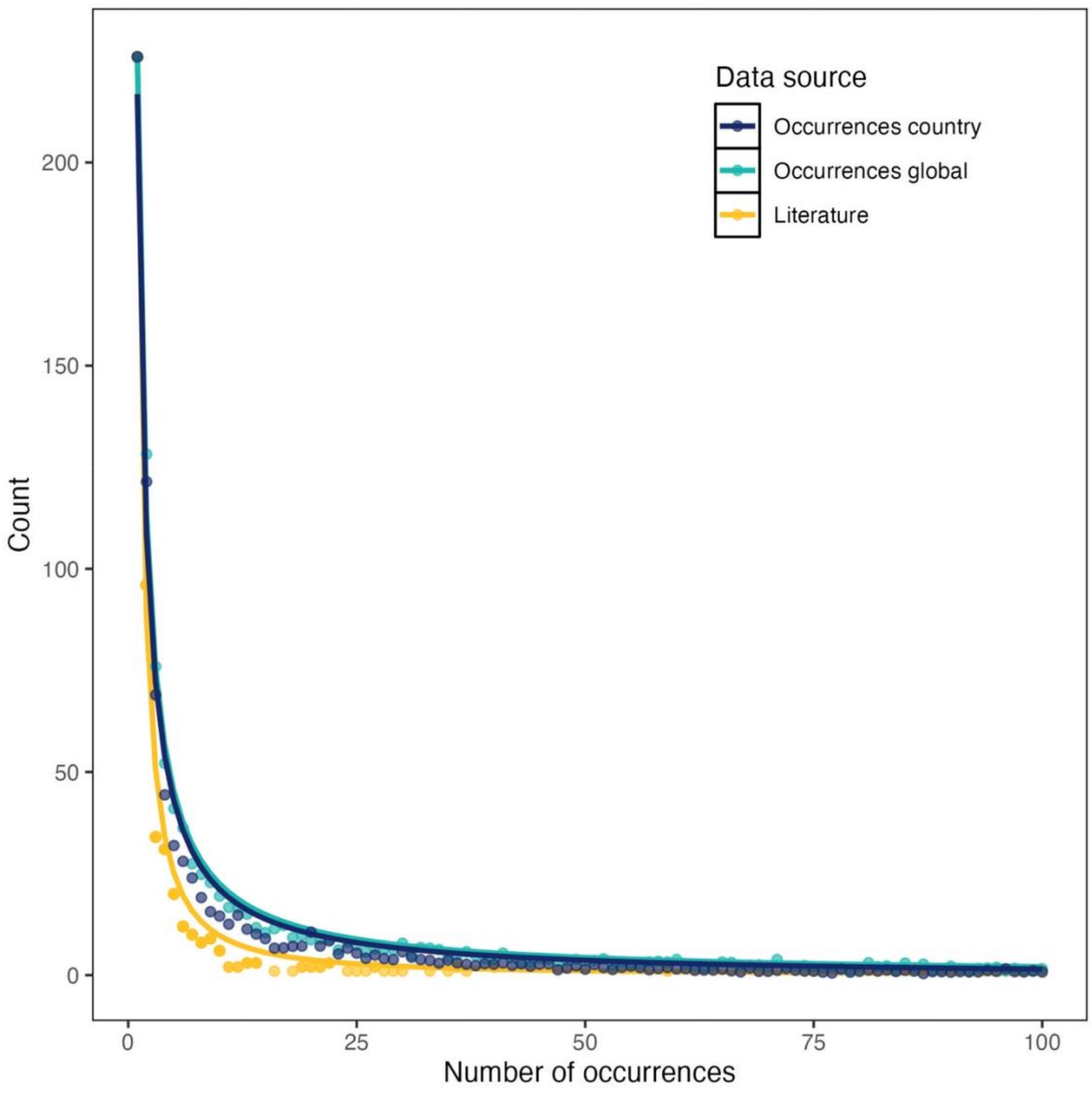

Fig. S10.

The curves (lines) generated from empirical data (points) on the number of specimens per species from three source. The yellow shows data from the most-recent taxonomic works for 497 species with a formula of $y = (226.6 \cdot x) \cdot x^{-\log(10.7)}$ (SE = 6.1). The light blue shows data derived from species counts at the global dataset level with a formula of $y = -0.1891 + 226.4555/x$ (SE = 0.6). The dark purple shows data derived from species counts at the country dataset level with a formula of $= -0.68833 + 217.51375/x$ (SE = 1.3). The original publication and original input data can be downloaded from https://doi.org/10.25451/flinders.21709757. Source data are provided as a Source Data file.

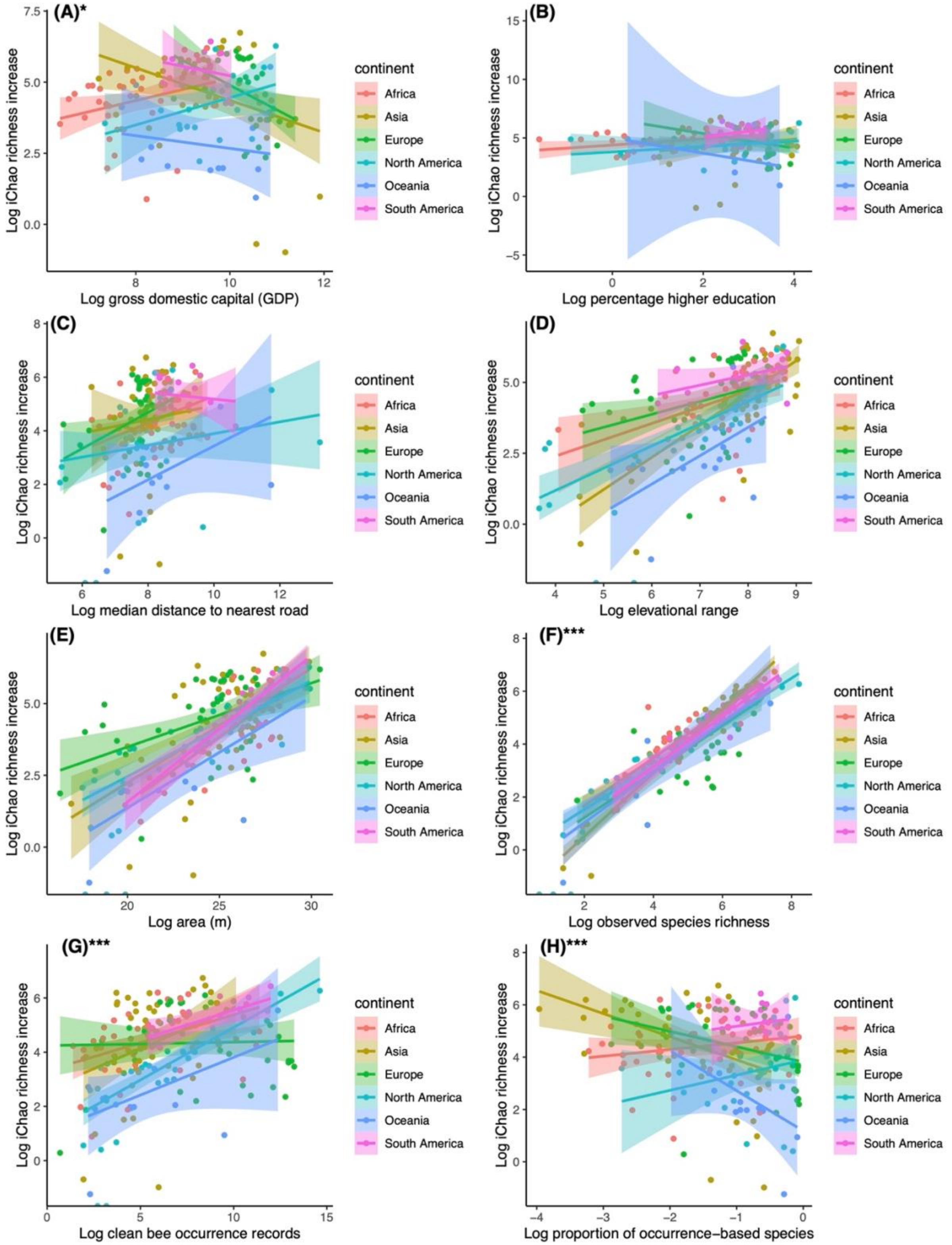

Fig. S11.

The linear relationships between the log of iChao species richness increase (Y-axis) and the log of (A) gross domestic product (GDP) per capita [42], (B) tertiary education [43], (C) country-wide median distance from roads [46], (D) elevational range [44], (E) the area of each country in meters, (F) the observed species richness, (G) the number of

clean BeeBDC records, and (H) the proportion of species derived from the literature curve. Asterisks indicate statistical significance in the overall model (* 0.5–0.1; ** >0.1–0.001; *** >0.001). The original publication and original input data can be downloaded from https://doi.org/10.25451/flinders.21709757. Source data are provided as a Source Data file.

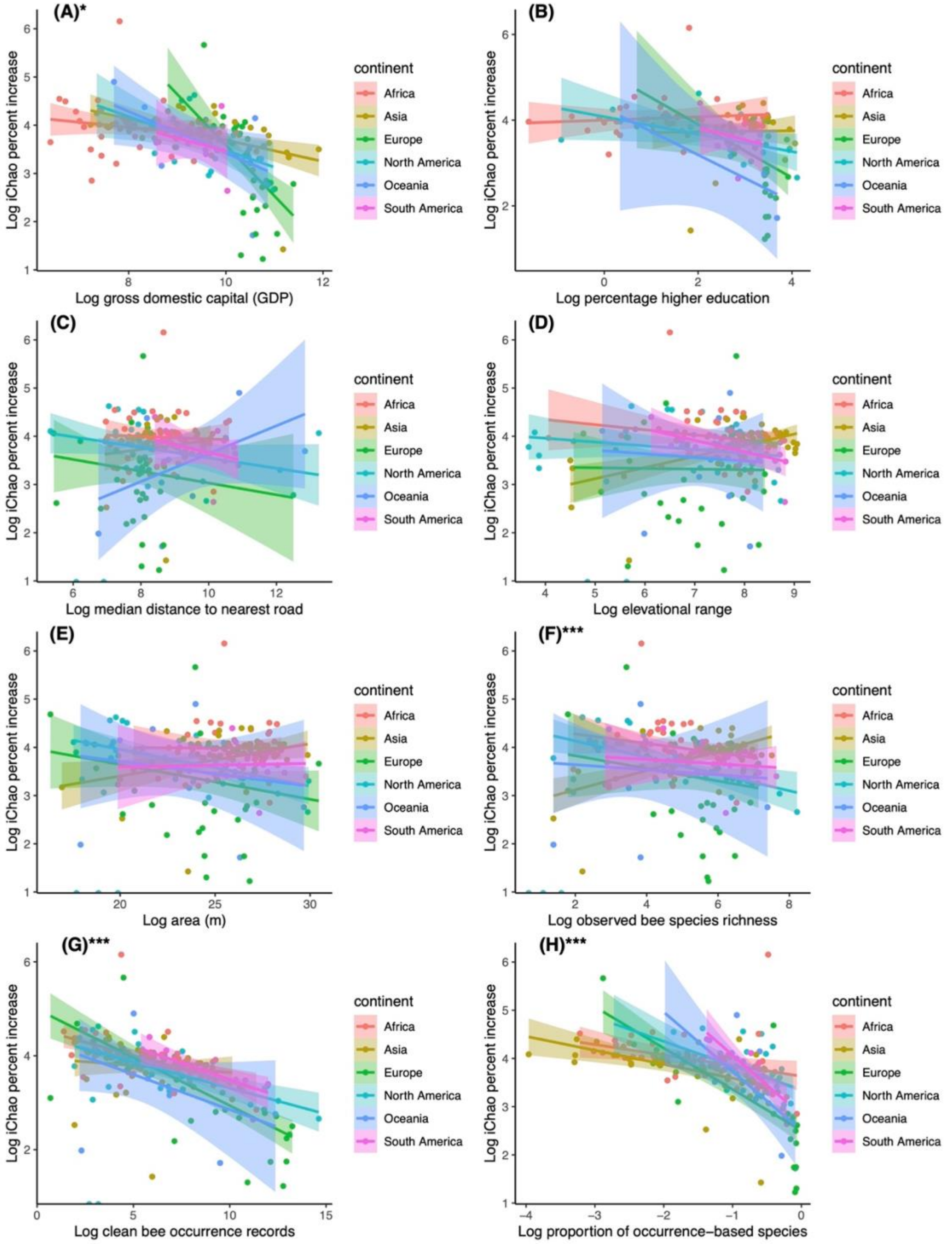

Fig. S12.

The linear relationships between the log of iChao percentage species richness increase (Y-axis) and the log of (A) gross domestic product per capita (GDPc) [42], (B) tertiary education [43], (C) country-wide median distance from roads

[46], (D) elevational range [44], (E) the area of each country in meters, (F) the observed species richness, (G) the number of clean BeeBDC records, and (H) the proportion of species derived from the literature curve. Asterisks indicate statistical significance in the overall model (* 0.5–0.1; ** >0.1–0.001; *** >0.001). The original publication and original input data can be downloaded from https://doi.org/10.25451/flinders.21709757. Source data are provided as a Source Data file.

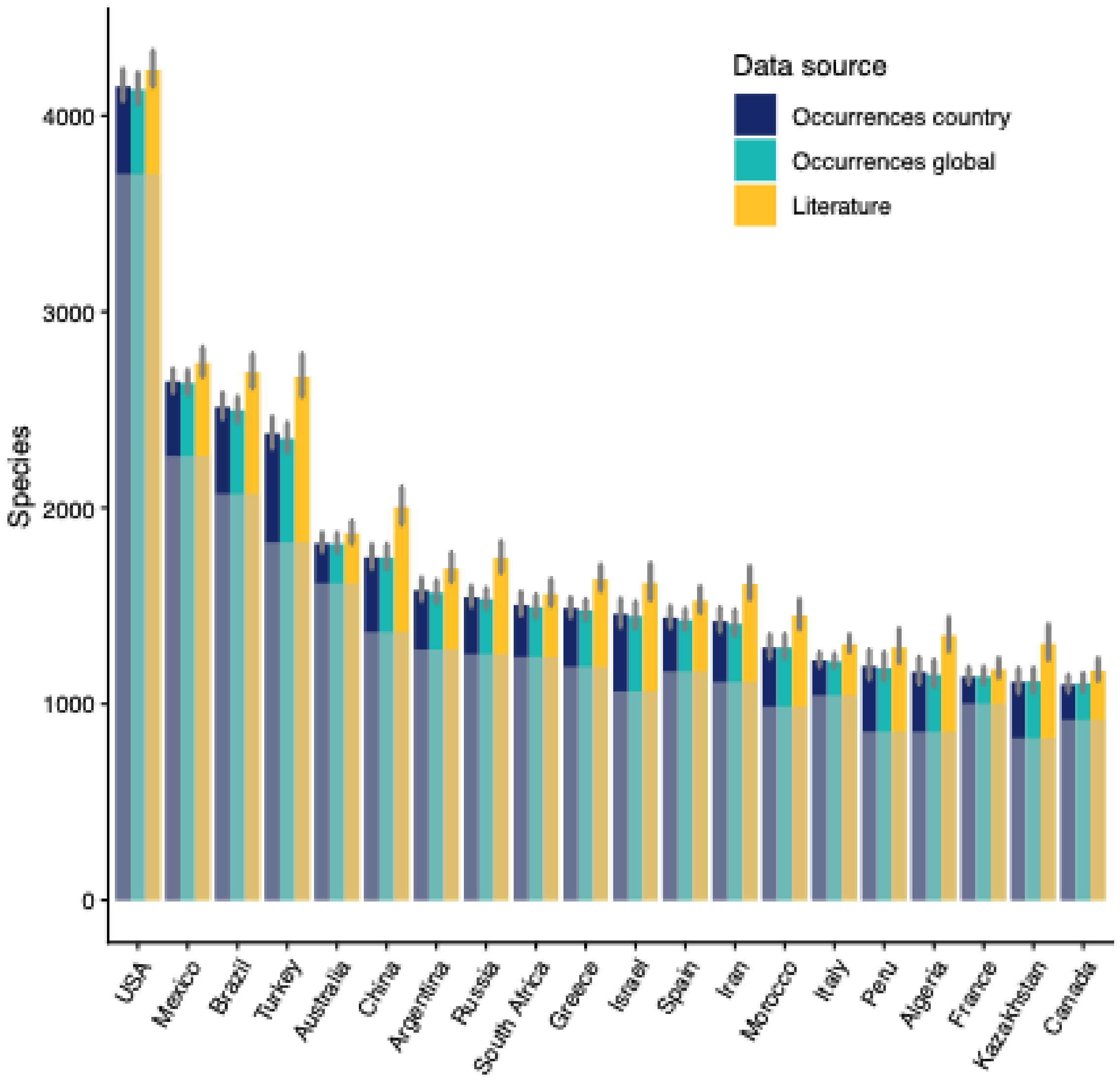

Fig. S13.

Bar plots of the top 20 countries predicted to have the highest species richness (according to the literature curve). For each country the number of observed species (grey) and the number of estimated species using the iChao statistic. A different value is shown depending on if the no-occurrence records were estimated from the global occurrence (light blue), country occurrence (dark purple) or literature (yellow) curves (Fig. S10). Bars indicate 95% confidence intervals. The original publication and original input data can be downloaded from https://doi.org/10.25451/flinders.21709757. Source data are provided as a Source Data file.

Table S1.

Australian taxonomy papers since the year 2000 that described new species or synonyms. Papers that did include phylogenetic analyses are marked as "TRUE" for that column.

Taxon	New species	New synonyms	Title	doi	Year	Journal	Authors	Phylogeny used
Callalictus	0	4	Taxonomic revision of the Australian native bee subgenus *Callalictus* (Hymenoptera: Halictidae: Halictini: genus *Lasioglossum*)	https://orcid.org/0000-0001-8750-2035	2022	Australian Journal of Taxonomy	Kenneth L. Walker	FALSE
Amegilla (Notomegilla), Amegilla (Zonamegilla)	0	31	The genus *Amegilla* (Hymenoptera, Apidae, Anthophorini) in Australia: A revision of the subgenera *Notomegilla* and *Zonamegilla*	https://doi.org/10.3897/zookeys.653.11177	2017	ZooKeys	Remko Leijs, Michael Batley, Katja Hogendoorn	TRUE
Austroplebeia	0	5	Australian and New Guinean Stingless Bees of the Genus *Austroplebeia* Moure (Hymenoptera: Apidae)—a revision	https://doi.org/10.11646/zootaxa.4047.1.1	2016	Zootaxa	Anne E. Dollin, Leslie J. Dollin & Claus Rasmussen	FALSE
Leioproctus (Exleycolletes)	2	0	New species in the bee subgenus *Leioproctus (Exleycolletes)* Maynard (Hymenoptera: Colletidae)	https://doi.org/10.54102/ajt	2023	Australian Journal of Taxonomy	Michael Batley	FALSE
Euhesma	3	0	A New Group of Euryglossine Bees from Australia (Hymenoptera: Apoidea: Colletidae)	http://www.jstor.org/stable/25086259 .	2004	Journal of the Kansas Entomological Society	Elizabeth M. Exley	FALSE
Euhesma	7	0	Bees of the *Euhesma crabronica* species-group (Hymenoptera: Colletidae: Euryglossinae)	https://museum.wa.gov.au/sites/default/files/BEES%20OF%20THE%20EUSHESMA%20CRABRONICA%20SPECIES-GROUPD%20(HYMENOPTERA%20COLLETI	2002	Records of the Western Australian Museum	Elizabeth M. Exley	FALSE

				FDAE%20EURYGLOSSINAE).pdf				
Euhesma	9	0	The walkeriana species-group of *Euhesma* Michener (Hymenoptera: Colletidae: Euryglossinae)	https://doi.org/10.1046/j.1440-6055.2001.00214.x	2001	Australian Journal of Entomology	Elizabeth M. Exley	FALSE
Exoneurella	1	0	Taxonomy of the Australian Allodapine Bee Genus *Exoneurella* (Apidae: Xylocopinae: Allodapini) and Description of a New *Exoneurella* Species	https://doi.org/10.1093/isd/ixx013	2018	Insect Systematics and Diversity	Rebecca M. Dew, Mark I. Stevens, And Michael P. Schwarz	TRUE
Goniocolletes	2	0	New species of *Goniocolletes* and *Trichocolletes* (Hymenoptera, Colletidae) from southern Australia	https://doi.org/10.3897/zookeys.598.9229	2016	ZooKeys	Remko Leijs, Katja Hogendoorn	FALSE
Leioproctus	33	59	Revision of *Goniocolletes* and seven Australian subgenera of *Leioproctus* (Hymenoptera: Apoidea: Colletidae), and description of new taxa	http://dx.doi.org/10.11646/zootaxa.3715.1.1	2014	Zootaxa	Glynn Vivian Maynard	FALSE
Austrothurgus	1	0	The lithurgine bees of Australia (Hymenoptera: Megachilidae), with a note on *Megachile rotundipennis*	http://dx.doi.org/10.17161/jom.v0i11.4520	2013	Journal of Melittology	Victor H. Gonzalez, Michael S. Engel, & Terry L. Griswold	FALSE
Euhesma	3	0	DNA barcoding of euryglossine bees and the description of new species of *Euhesma* Michener (Hymenoptera, Colletidae, Euryglossinae)	http://dx.doi.org/10.3897/zookeys.520.6185	2015	ZooKeys	Katja Hogendoorn, Mark Stevens, Remko Leijs	TRUE
Inquilina	5	0	New species and unexpected diversity of socially parasitic bees in the genus *Inquilina* Michener (Hymenoptera: Apoidea: Apidae)	https://doi.org/10.1111/j.1744-7917.2009.01266.x	2009	Insect Science	Jaclyn A. Smith And Michael P. Schwarz	TRUE

Leioproctus (Colletellus)	26	0	Twenty-six new species of *Leioproctus (Colletellus)*: Australian Neopasiphaeinae, all but one with two submarginal cells (Hymenoptera, Colletidae, *Leioproctus*)	https://doi.org/10.3897/zookeys.811.28924	2018	ZooKeys	Remko Leijs, James Dorey, Katja Hogendoorn	FALSE
Leioproctus (Protomorpha)	5	0	Five new species of *Leioproctus (Protomorpha)* Rayment (Hymenoptera: Colletidae)	http://dx.doi.org/10.3853/j.2201-4349.65.2013.1597	2013	Australian Museum Scientific Publications	Batley, Michael, And Tony J. Popic	FALSE
Paracolletinae	1	0	A new *Leioproctus* with unique wing venation in males (Hymenoptera: Colletidae: Paracolletinae) with comments on unusual wing modifications in bees	https://doi.org/10.11646/zootaxa.1104.1.4	2006	Zootaxa	Laurence Packer	FALSE
Coelioxys	2	3	A revision of the cleptoparasitic bee genus *Coelioxys* (Hymenoptera: Megachilidae) from Australia	http://dx.doi.org/10.14411/eje.2016.002	2016	European Journal of Entomology	Léo Correia Da Rocha-Filho	FALSE
Trichocolletes	23	4	Revision of the Australian bee genus *Trichocolletes* Cockerell (Hymenoptera: Colletidae: Paracolletini)	http://dx.doi.org/10.3853/j.0067-1975.64.2012.1589	2012	Australian Museum Scientific Publications	Batley, Michael, And Terry F. Houston	FALSE
Parasphecodes	18	69	Taxonomic revision of the native bee subgenus *Parasphecodes* Smith 1853 in Australia (Hymenoptera: Halictidae: Halictini: *Lasioglossum* Curtis 1833)	https://doi.org/10.11646/zootaxa.5408.1.1	2024	Zootaxa	Kenneth L. Walker & Kathryn S. Sparks	FALSE
Australictus	0	6	Taxonomic revision of the Australian native bee subgenus *Australictus* (Hymenoptera: Halictidae: Halictini: genus *Lasioglossum*) – “Wood-Splitting Axe Bees”	https://doi.org/10.24199/j.mmv.2022.81.06	2022	Memoirs of Museum Victoria	Kenneth L. Walker	FALSE

Koptortosoma	2	3	A revision of the Australian carpenter bees, genus *Xylocopa* Latreille, subgenera *Koptortosoma* Gribodo and *Lestis* Lepeletier & Serville (Hymenoptera : Apidae)	https://doi.org/10.1071/IT98014	2000	Invertebrate Taxonomy	Remko Leijs	FALSE
Amegilla (Asaropoda)	10	16	The genus *Amegilla* (Hymenoptera, Apidae, Anthophorini) in Australia: a revision of the subgenus *Asaropoda*	https://doi.org/10.3897/zookeys.908.47375	2020	Zookeys	Remko Leijs, James Dorey, Katja Hogendoorn	TRUE

Table S2.

The outputs from our two-sided linear mixed-effects models of the increased number of undescribed species (iChao) per country with the random effect of continent and several variables. We included one interaction term between the log of clean records and the log proportion of species derived from the literature curve. Bolded values indicate statistical significance.

Variable	Estimate	SE	df	t-value	Pr(>|t|)
(Intercept)	0.84	0.76	114.21	1.10	0.27
log(GPD_per_cap)	-0.14	0.06	111.00	-2.52	**0.01**
log(percent_ed)	0.07	0.05	115.99	1.56	0.12
log(median_roadDist)	-0.02	0.06	115.00	-0.38	0.71
log(elevationalRange)	0.02	0.05	88.67	0.46	0.65
log(area_m)	0.00	0.03	108.83	-0.09	0.93
log(observedRichness)	1.30	0.07	98.31	19.38	**< 2e-16**
log(cleanRecords)	-0.29	0.04	115.83	-7.88	**2.00E-12**
log(propOccurrences)	0.43	0.10	115.38	4.21	**5.04E-05**
log(cleanRecords):log(propOccurrences)	-0.05	0.02	111.84	-2.55	**0.01**

Table S3.

The outputs from our two-sided linear mixed-effects models of the percentage increase in the undescribed species (iChao) per country with the random effect of continent and several variables. We included one interaction term between the log of clean records and the log proportion of species derived from the literature curve. Bolded values indicate statistical significance. Standard error (SE), degrees of freedom (df), and the p-value (Pr(>|t|)) are provided.

**Variable**	**Estimate**	**SE**	**df**	**t-value**	**Pr(>|t|)**
(Intercept)	5.44	0.76	114.21	7.12	**1.04E-10**
log(GPD_per_cap)	-0.14	0.06	111.00	-2.52	**0.01**
log(percent_ed)	0.07	0.05	115.99	1.56	0.12
log(median_roadDist)	-0.02	0.06	115.00	-0.38	0.71
log(elevationalRange)	0.02	0.05	88.67	0.46	0.65
log(area_m)	0.00	0.03	108.83	-0.09	0.93
log(observedRichness)	0.30	0.07	98.31	4.51	**1.78E-05**
log(cleanRecords)	-0.29	0.04	115.83	-7.88	**2.00E-12**
log(propOccurrences)	0.43	0.10	115.38	4.21	**5.04E-05**
log(cleanRecords):log(propOccurrences)	-0.05	0.02	111.84	-2.55	**0.01**

Table S4.

The outputs from our two-sided linear mixed-effects models of the number and percentage increase in the undescribed species (iChao) per country with the random effect of continent and several variables. The table contains four analyses alternating between the number increase and percentage increase. The analyses fill in the no-record species with data drawn from the (a) global species occurrence dataset or (b) country-level occurrence dataset. We included one interaction term between the log of clean records and the log proportion of species derived from the literature curve. Bolded values indicate statistical significance. Standard error (SE), degrees of freedom (df), and the p-value (Pr(>|t|)) are provided.

Variable	Estimate	SE	df	t-value	Pr(>|t|)
Global iChao increase					
(Intercept)	0.56	0.86	113.47	0.65	0.52
log(GPD_per_cap)	-0.11	0.06	110.86	-1.71	0.09
log(percent_ed)	0.05	0.05	114.99	0.93	0.36
log(median_roadDist)	-0.03	0.07	113.97	-0.39	0.70
log(elevationalRange)	0.02	0.06	88.02	0.41	0.68
log(area_m)	0.00	0.03	108.62	0.12	0.91
log(observedRichness)	1.27	0.08	101.25	16.46	**< 2e-16**
log(cleanRecords)	-0.31	0.04	114.70	-7.41	**2.33E-11**
log(propOccurrences)	0.56	0.11	114.53	4.86	**3.72E-06**
log(cleanRecords):log(propOccurrences)	-0.04	0.02	111.35	-1.92	0.06
**Global iChao percentage**					
(Intercept)	5.17	0.86	113.47	6.03	**2.14E-08**
log(GPD_per_cap)	-0.11	0.06	110.86	-1.71	0.09
log(percent_ed)	0.05	0.05	114.99	0.93	0.36
log(median_roadDist)	-0.03	0.07	113.97	-0.39	0.70
log(elevationalRange)	0.02	0.06	88.02	0.41	0.68
log(area_m)	0.00	0.03	108.62	0.12	0.91
log(observedRichness)	0.27	0.08	101.25	3.53	**6.24E-04**
log(cleanRecords)	-0.31	0.04	114.70	-7.41	**2.33E-11**
log(propOccurrences)	0.56	0.11	114.53	4.86	**3.72E-06**
log(cleanRecords):log(propOccurrences)	-0.04	0.02	111.35	-1.92	0.06
**Country iChao increase**					
(Intercept)	0.51	0.83	114.62	0.61	0.54
log(GPD_per_cap)	-0.10	0.06	113.38	-1.55	0.12
log(percent_ed)	0.04	0.05	114.77	0.87	0.39
log(median_roadDist)	-0.04	0.06	114.71	-0.69	0.49
log(elevationalRange)	0.03	0.06	100.90	0.47	0.64
log(area_m)	0.01	0.03	112.17	0.26	0.80
log(observedRichness)	1.26	0.08	107.88	16.74	**< 2e-16**
log(cleanRecords)	-0.30	0.04	114.98	-7.54	**1.19E-11**
log(propOccurrences)	0.56	0.11	113.96	5.03	**1.86E-06**
log(cleanRecords):log(propOccurrences)	-0.05	0.02	114.00	-2.11	**0.04**

Country iChao percentage					
(Intercept)	5.11	0.83	114.62	6.15	**1.14E-08**
log(GPD_per_cap)	-0.10	0.06	113.38	-1.55	0.12
log(percent_ed)	0.04	0.05	114.77	0.87	0.39
log(median_roadDist)	-0.04	0.06	114.71	-0.69	0.49
log(elevationalRange)	0.03	0.06	100.90	0.47	0.64
log(area_m)	0.01	0.03	112.17	0.26	0.80
log(observedRichness)	0.26	0.08	107.88	3.45	**7.98E-04**
log(cleanRecords)	-0.30	0.04	114.98	-7.54	**1.19E-11**
log(propOccurrences)	0.56	0.11	113.96	5.03	**1.86E-06**
log(cleanRecords):log(propOccurrences)	-0.05	0.02	114.00	-2.11	**0.04**
