# Supplementary material for: Estimating global bee species richness and taxonomic gaps
Source: Nat Commun. 2026 Feb 24;17:1762. doi: 10.1038/s41467-026-69029-4 (PMC12932799; doi:10.1038/s41467-026-69029-4)
Supplement: Supplementary file 2 — Reporting Summary [file 41467_2026_69029_MOESM2_ESM.pdf]

Corresponding author(s): James Dorey and Michael Orr

Last updated by author(s): Jan 7, 2026

Reporting Summary

Nature Portfolio wishes to improve the reproducibility of the work that we publish. This form provides structure for consistency and transparency in reporting. For further information on Nature Portfolio policies, see our Editorial Policies and the Editorial Policy Checklist.

Statistics

For all statistical analyses, confirm that the following items are present in the figure legend, table legend, main text, or Methods section.

n/a	Confirmed	
☐	☒	The exact sample size (*n*) for each experimental group/condition, given as a discrete number and unit of measurement
☐	☒	A statement on whether measurements were taken from distinct samples or whether the same sample was measured repeatedly
☐	☒	The statistical test(s) used AND whether they are one- or two-sided<br>*Only common tests should be described solely by name; describe more complex techniques in the Methods section.*
☐	☒	A description of all covariates tested
☐	☒	A description of any assumptions or corrections, such as tests of normality and adjustment for multiple comparisons
☐	☒	A full description of the statistical parameters including central tendency (e.g. means) or other basic estimates (e.g. regression coefficient) AND variation (e.g. standard deviation) or associated estimates of uncertainty (e.g. confidence intervals)
☐	☒	For null hypothesis testing, the test statistic (e.g. *F*, *t*, *r*) with confidence intervals, effect sizes, degrees of freedom and *P* value noted<br>*Give P values as exact values whenever suitable.*
☒	☐	For Bayesian analysis, information on the choice of priors and Markov chain Monte Carlo settings
☒	☐	For hierarchical and complex designs, identification of the appropriate level for tests and full reporting of outcomes
☒	☐	Estimates of effect sizes (e.g. Cohen's *d*, Pearson's *r*), indicating how they were calculated

Our web collection on statistics for biologists contains articles on many of the points above.

Software and code

Policy information about availability of computer code

Data collection	All code used to collect data is described in the manuscript. Where data were collected using code, it was done using BeeBDC version 1.2.0. Other datasets were downloaded from publicly available websites and these are also cited in-text.
Data analysis	All code, including versions, are explicitly stated in text. All novel code has been made available and is additionally publicly available on CRAN as a new version of BeeBDC (1.3.1).

For manuscripts utilizing custom algorithms or software that are central to the research but not yet described in published literature, software must be made available to editors and reviewers. We strongly encourage code deposition in a community repository (e.g. GitHub). See the Nature Portfolio guidelines for submitting code & software for further information.

Data

Policy information about availability of data

All manuscripts must include a data availability statement. This statement should provide the following information, where applicable:

- Accession codes, unique identifiers, or web links for publicly available datasets
- A description of any restrictions on data availability
- For clinical datasets or third party data, please ensure that the statement adheres to our policy

The core functions required to complete these analyses for bees or other taxa are available with BeeBDC v.1.3.1 or higher (https://github.com/jbdorey/BeeBDC). A vignette of these functions is available at https://jbdorey.github.io/BeeBDC/articles/speciesRichness_example.html. Species occurrence, taxonomy, and checklist data are all available from Flinders ROADS (FigShare) at https://doi.org/10.25451/flinders.21709757. The latter two datasets are also available through and updated

in the BeeBDC R package and formatted taxonomy files for other taxa can be downloaded using BeeBDC::taxadbToBeeBDC. Our R scripts are all available on GitHub (https://github.com/jbdorey/BDE_R_wofklow) along with input and interim files, tables, figures, and additional files referred to throughout. All source data for figures has been uploaded as Supplementary Data.

Research involving human participants, their data, or biological material

Policy information about studies with human participants or human data. See also policy information about sex, gender (identity/presentation), and sexual orientation and race, ethnicity and racism.

Reporting on sex and gender	No human sex or gender data are included in our manuscript.
Reporting on race, ethnicity, or other socially relevant groupings	No race, ethnicity, or social-relevant grouping data are used. Some socio-economic variables are used, and cited within the body of text, in order to find correlations for taxonomic impediments.
Population characteristics	NA
Recruitment	NA
Ethics oversight	NA

Note that full information on the approval of the study protocol must also be provided in the manuscript.

Field-specific reporting

Please select the one below that is the best fit for your research. If you are not sure, read the appropriate sections before making your selection.

☐ Life sciences ☐ Behavioural & social sciences ☒ Ecological, evolutionary & environmental sciences

For a reference copy of the document with all sections, see nature.com/documents/nr-reporting-summary-flat.pdf

Ecological, evolutionary & environmental sciences study design

All studies must disclose on these points even when the disclosure is negative.

Study description	Our study uses publicly available species occurrence datasets from several sources (online repositories, published papers, published datasets...). We extend existing non-parametric statistical estimates of species richness at the global, continental, and country levels in order to estimate the number of bee species at each of those levels. We run further checks of these outcomes using species accumulation rates, random sampling of the input data, and checking perturbations to the input formula and data.
Research sample	We used a global, cleaned, and publicly available and published (Scientific Data) dataset of >8 million bee occurrence records from around the world. These data are sourced from BeeBDC and originally from several data repositories and smaller providers. Additional data are sourced from published manuscripts.
Sampling strategy	Sampling was based on the available data around the world. Countries with a sample size of <30, or where estimates were an order of magnitude or more larger than empirical the size were excluded. Because our methods allow the estimation of uncertainty, we were able to otherwise leave countries with large uncertainty in our analyses, but with those error margins clear in all figures and tables.
Data collection	Most data were downloaded from the BeeBDC dataset. Some data were extracted from published papers; either from a literature search on Scopus, by searching for the most-recent taxonomic work randomly-selected species, or from known publicly-available datasets. These searches were undertaken by all authors.
Timing and spatial scale	Our data range across the whole sampling/taxonomic history of bee research (~1755 to 2024). Data are spread across this time period and are taken across the globe.
Data exclusions	We filtered the uncleaned version of the BeeBDC dataset to remove records with (i) invalid binomials, (ii) improper basis of record, (iii) unmatched coordinates and country name, (iv) absences, (v) invalid license, or (vi) outside of their country checklist using BeeBDC. These filters were considered relevant for the current manuscript which required at least country and species level data.
Reproducibility	All of our major analyses were undertaken 100 times per level and were further analysed using different input formula to test for the sensitivity of the analyses. All iterations were "successful".
Randomization	We did not randomize our groups as this was not relevant. However, we did randomly sample from an empirical distribution in order to fill data gaps and account for the known species richness in particular regions.
Blinding	All data and analyses were collected and conducted blindly and a priori.

Did the study involve field work? ☐ Yes ☒ No

Reporting for specific materials, systems and methods

We require information from authors about some types of materials, experimental systems and methods used in many studies. Here, indicate whether each material, system or method listed is relevant to your study. If you are not sure if a list item applies to your research, read the appropriate section before selecting a response.

Materials & experimental systems

n/a	Involved in the study	
☒	☐	Antibodies
☒	☐	Eukaryotic cell lines
☒	☐	Palaeontology and archaeology
☐	☒	Animals and other organisms
☒	☐	Clinical data
☒	☐	Dual use research of concern
☒	☐	Plants

Methods

n/a	Involved in the study	
☒	☐	ChIP-seq
☒	☐	Flow cytometry
☒	☐	MRI-based neuroimaging

Animals and other research organisms

Policy information about studies involving animals; ARRIVE guidelines recommended for reporting animal research, and Sex and Gender in Research

Laboratory animals	NA
Wild animals	NA
Reporting on sex	NA
Field-collected samples	NA
Ethics oversight	Insects do not require animal ethics and no live insects were included in our analyses.

Note that full information on the approval of the study protocol must also be provided in the manuscript.

Plants

Seed stocks	NA
Novel plant genotypes	NA
Authentication	NA
